# Parasites of Moroccan desert *Coptodon guineensis* (Pisces, Cichlidae): transition and resilience in a simplified hypersaline ecosystem[Fn FN1]

**DOI:** 10.1051/parasite/2022064

**Published:** 2022-12-23

**Authors:** Halima Louizi, Kristina M. Hill-Spanik, Abdeljebbar Qninba, Vincent A. Connors, Amine Belafhaili, Jean-Francois Agnèse, Antoine Pariselle, Isaure de Buron

**Affiliations:** 1 Laboratory Biodiversity, Ecology and Genome, Mohammed V University in Rabat, Faculty of Sciences 10000 Rabat Morocco; 2 Department of Biology, College of Charleston 205 Fort Johnson Road Charleston SC 29412 USA; 3 Mohammed V University in Rabat, Institut Scientifique, Avenue Ibn Batouta B.P. 703 10090 Agdal Rabat Morocco; 4 Division of Natural Sciences, University of South Carolina-Upstate 800 University Way Spartanburg SC 29303 USA; 5 LMNE, Mohammed V University in Rabat, Faculty of Sciences 10000 Rabat Morocco; 6 ISEM, CNRS, Université de Montpellier, IRD, EPHE 34095 Montpellier France

**Keywords:** Acanthocephala, Metacercaria, *Acanthogyrus* (*Acanthosentis*) *tilapiae*, *Pygidiopsis genata*, Host-switching, Sebkha Imlili, Sahara, Wetland

## Abstract

Sebkha Imlili (Atlantic Sahara) is a salt flat with over 160 permanent holes of hypersaline water generated in the Holocene and inhabited by euryhaline organisms that are considered to be relics of the past, including the cichlid fish *Coptodon guineensis*. We surveyed the fish parasites four times over one year, to i) identify the parasites, and ii) determine possible seasonality in infection patterns. Over 60% of the fish were infected by one to three helminths: an acanthocephalan in the intestine and two digenean metacercariae in the kidney, spleen, liver, muscle, and mesenteries. The acanthocephalan *Acanthogyrus* (*Acanthosentis*) cf. *tilapiae* was identified morphologically and molecularly; only one digenean (the heterophyid *Pygidiopsis genata*) could be identified molecularly. Both identified parasites were present throughout the sampling periods; the unidentified metacercariae were present only in summer and fall. Mean intensities, but not prevalence of infection by the acanthocephalan, reflected a biannual pattern of transmission. Infection accrued with fish size, possibly due to cannibalism. Because the water holes include only a few invertebrates, the intermediate hosts of these parasites can be inferred to be the gastropod *Ecrobia ventrosa* for the digeneans and either the copepod *Cletocamtpus retrogressus* or the ostracod *Cyprideis torosa* for the acanthocephalan. This ecosystem appears stable and provides a window into the past, as the acanthocephalan likely switched from freshwater tilapia to *C. guineensis* when the Sebkha formed. However, this is a vulnerable environment where the survival of these parasites depends on interactions maintained among only very few hosts.

## Abstract (fr)

La Sebkha d’Imlili est une sebkha (étendue désertique sableuse et salée) dans le Sahara Atlantique caractérisée par la présence de plus de 160 poches permanentes d’eau hypersaline qui sont apparues à l’Holocène et qui sont habitées par des organismes considérés comme des reliques du passé, dont un poisson cichlidé, *Coptodon guineensis*. Nous avons fait l’inventaire des parasites de ce poisson au cours des quatre saisons d’une année pour 1) identifier les parasites et 2) déterminer une éventuelle transmission saisonnière. Plus de 60 % des poissons étaient infestés par un à trois helminthes : un Acanthocéphale dans l’intestin et des métacercaires de deux espèces de Digène dans le rein, la rate, les muscles et le mésentère. L’Acanthocéphale *Acanthogyrus (Acanthosentis)* cf. *tilapiae* a été identifié morphologiquement et génétiquement mais seul un des deux Digènes (l’hétérophyidé *Pygidiopsis genata*) a pu être identifié par séquençage. Ces deux parasites étaient présents à chaque période d’étude, mais la métacercaire non identifiée était présente seulement en été et en automne. L’intensité moyenne de l’infestation par l’Acanthocéphale, mais pas sa prévalence, reflète une transmission biannuelle. L’infestation augmente avec la taille du poisson, peut-être à cause du cannibalisme. L’identité des hôtes intermédiaires de ces parasites peut être avancée parce que cet écosystème est simplifié et inclut seulement quelques invertébrés : pour les Digènes, le Gastéropode *Ecrobia ventrosa*, et pour l’Acanthocéphale, le Copépode *Cletocamtpus retrogressus* ou l’Ostracode *Cyprideis torosa*. Cet écosystème apparait stable et offre une vue sur le passé étant donné que l’Acanthocéphale a sans doute été transféré d’un tilapia d’eau douce quand la sebkha s’est formée. Cependant, c’est un environnement vulnérable où la survie de ces parasites dépend d’interactions entre très peu d’espèces hôtes.

## Introduction

The Sebkha of Imlili is a unique Saharan wetland belonging to the Meso-Cenozoic basin of Tarfaya-Laâyoune-Dakhla located in the extreme south of Morocco, approximately 50 km south of Dakhla and about 15 km from the Atlantic Ocean [46]. Since 2018, the Sebkha has been classified as a wetland site of international importance under the Ramsar Convention as a significant waterfowl habitat. It is distinguished from other sebkhas by its physiognomy, its hydrological functioning, and its biological diversity [85]. The Sebkha Imlili is an endorheic depression, elongate (~13 km long by 2.5 km wide), and generally oriented NNE-SSW [84]. It is surrounded by a sandy dune formation, upon which fairly dense desert-type vegetation develops. In the northern part of the Sebkha, the sandy soil is brick red and covered, in the driest areas, with a fine whitish powder of crystallized salt. The Sebkha is unique because of the presence in its northern part of more than 160 holes (or pools) of permanent saline to hypersaline water, the sustainability of which is ensured by resurgences of the superficial groundwater table, itself fed by occasional flooding in the region [37, 46]. These permanent pools vary in shape, diameter (1–10 m), volume (0.03–740 m^3^), and depth (~0.1–6 m). Pool bottoms are sandy, and their edges have concretions of sand and salt. The salinity of the water in these pockets ranges from 39 to 71 [71]. The lithological nature of Sebkha Imlili and neighboring outcrops influence the chemistry of the waters in the pockets. In short, the area around the depression, as well as the bottom of the Sebkha, are drained by “chaâbas” (streambeds that only flow during intense rainy episodes generating floods) that carry evaporites and reddish saliferous silty deposits whose salt loaded contents influence the salinity of the waters in the pockets [46].

Sebkha Imlili belongs to the coastal Sahara in an area where the average temperature varies between 5 °C in winter and 48 °C in summer. Rainfall is typically between 50 and 60 mm per year as the result of brief, violent, and irregular storms. These low rainfall amounts, as well as fog and dew, are sufficient to maintain semi-desert type vegetation in the Sebkha [88]. The area exhibits a great richness in terms of biodiversity, with recent studies providing new data on its reptiles [69], mammals [83], birds [81, 86], and flora [51]. On the other hand, the diversity of the aquatic fauna is relatively simple, with three species of crustaceans, three species of gastropod mollusks [40], and a single cichlid fish, *Coptodon guineensis* (Günther, 1862) [2, 48, 84]. Because these organisms are believed to have been trapped in the water pools when these were formed during the Holocene after the Green Sahara period, these aquatic animals are considered to be relics of the past [37].

Given that parasites in general, particularly specialists with narrow host ranges, are an integral part and drivers of biodiversity [38], the objective of our study was i) to identify the parasites of *C. guineensis* in the Sebkha in order to understand their origin (marine or freshwater), and ii) to obtain baseline data regarding the population dynamics of the identified parasites in order to understand the role they play in ecosystem function for future studies.

## Material and methods

### Fish sampling and parasite collection

**Figure 1:**
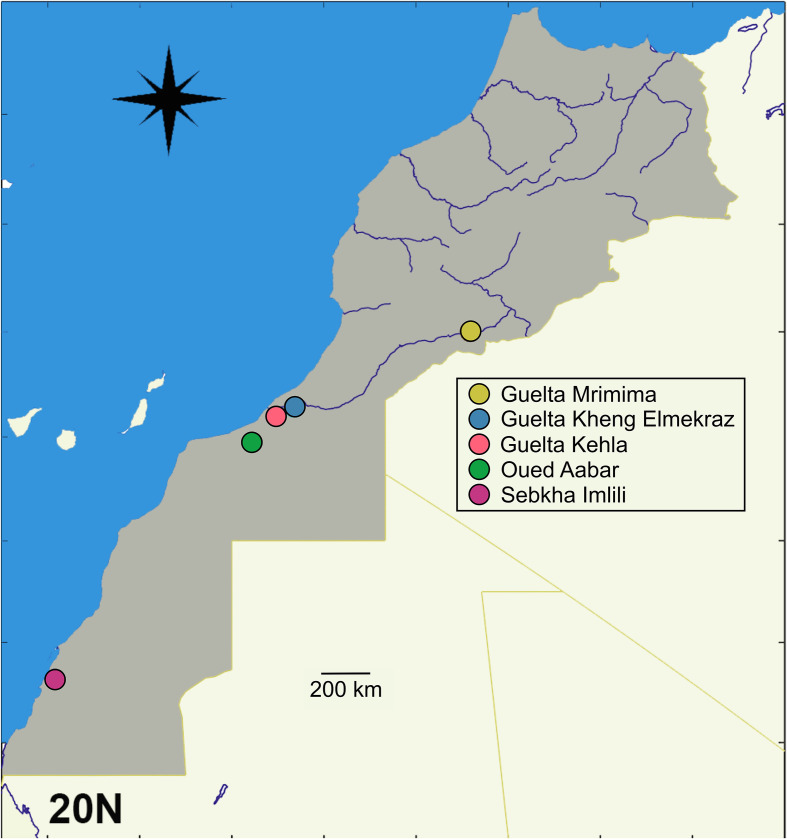
Map of Morocco indicating the sampling sites.

Specimens of *C. guineensis* (*N* = 322) were sampled using gillnets in two holes (#35: 23°16′35.21″ N, 15°54′55.47″ W and #121: 23°16′21.35″ N, 15°55′17.42″ W) in the Sebkha four times over one year: December 2018 and April, July, and October 2019, roughly reflecting winter, spring, summer, and fall seasons. Water temperatures and salinity in our reference water hole (#35) were 19 °C, 22 °C, 25 °C, 24 °C and 44, 42, 45, 35, respectively. Fish were measured (total length (TL) to the nearest mm) and sexed. TL averaged 86 ± 27 mm (range 17–181 mm). Other individuals of *C. guineensis* were also sampled further north in Oued Aabar (27°56′09.9″ N, 11°25′24.1″ W; Fig. 1). To compare parasite fauna with tilapia of other species, we also sampled redbelly tilapia *Coptodon zillii* (Gervais, 1848) (*N* = 2; TL = 200 mm) and blue tilapia *Oreochromis aureus* (Steindachner, 1864) (*N* = 12; TL = 158 ± 35 mm; range: 110–240 mm) from three gueltas (groundwater resurgences along dry wadi courses): one in the middle-Drâa (Guelta Mrimima: 29°49′23.642″ N, 006°58′36.12″ W) and two at the lower Drâa watershed (Guelta Kehla: 28°26′60″ N, 10°51′35.999″ W and Guelta Kheng Elmekraz: 28°22′23.92″ N, 10°22′52.28″ W) (Fig. 1). Fish from the gueltas were photographed, their TL measured, and a piece of the pectoral fin removed and stored in 96% ethanol (EtOH) for subsequent molecular identification [62]. Specimens of *O. aureus* are kept at the Laboratory of Biodiversity, Ecology and Genome at the University in Rabat, Morocco, three specimens of *C. guineensis* from the Sebkha Imlili are deposited at the Scientific Institute of Rabat under the numbers MNHN ZD1 01 17-a, MNHN ZD1 01 17-b, and MNHN ZD1 01-c, four specimens are deposited at the Royal Museum for Central Africa (RMCA 2022.020.P.0001; RMCA 2022.020.P.0002; RMCA 2022.020.P.0003; RMCA 2022.020.P.0004) and three others at the Royal Belgian Institute of Natural Sciences (RBINS 952; RBINS 953; RBINS 954). Sequences of *C. guineensis* were deposited in GenBank from a previous study [62] (Sebkha Imlili: MG755500; MG755474 and Oued Aabar: MK955801). Some fish were dissected fresh in the field, and others were frozen prior to dissection. Collection of the parasites was carried out by examination of the intestine and the body cavity under a dissecting microscope, and squashes of kidney, mesentery, spleen, gonads and skeletal muscles of the fish under a compound microscope. Gills were also examined under a dissecting microscope for a subsample of fish (*N* = 132). When fish were dissected fresh, acanthocephalans were relaxed in bottled (drinking) water for about 15 min at ambient temperature prior to fixation. Some metacercariae were excysted and heat fixed by passing a flame under the slide prior to fixation. Fixatives were 5% Neutral Buffered Formalin (NBF) for scanning electron microscopy and voucher preparation, and 96% EtOH for molecular identification or light microscopy. Vouchers of parasites were deposited at the Museum National d’Histoire Naturelle, Paris, France under the numbers MNHN HEL1881 – HEL1888.

### Light and scanning electron microscopy (SEM)

Acanthocephalans fixed in 96% EtOH were rinsed in water and examined in wet mounts to determine the sex ratio and stage of maturity of females. Measurements of morphological features were taken using a microscope (Leica DM 2500) equipped with a digital camera (Leica DMC 4500) and LAS version 4.12.0 software (all from Leica Microsystems GmbH, Wetzlar, Germany). A subset of specimens fixed in NBF was stained in acetocarmine or Meyer’s hematoxylin, dehydrated in a series of EtOH, and mounted in Canada balsam or Kleermount. For SEM, acanthocephalans and excysted metacercariae fixed in NBF were dehydrated in an EtOH series and chemically dried overnight using hexamethyldisilazane (HMDS), coated with gold using a sputter coater (JEOL JFC-2300HR, Japan), and observed with a SEM JEOL JSM IT 100 (Japan) under 10 kV. Measurements are means (range; number of specimens observed) given in μm unless otherwise stated. Proboscises were measured from tip to anterior limit of neck; hook blades were measured from point to top anterior surface. Vouchers of *Acanthogyrus* (*Acanthosentis*) *tilapiae* Baylis, 1947 deposited at the Smithsonian Institution National Museum of Natural History, USA were examined, specifically USNM 1369857, 1383211-1383227, 1394959 [10]*.*

### Molecular study

DNA from parasites fixed in 96% EtOH was isolated using a DNeasy Blood and Tissue kit (Qiagen, Germantown, MA, USA), following the manufacturer’s protocol except for decreasing the elution volume to 100 μL. Based on the results of the morphological analysis for the acanthocephalan, we chose three nuclear markers for molecular identification of parasites based on sequences available in GenBank (Table 1): portions of the large (28S) and small (18S) subunit and the internal transcribed spacer (ITS) region of the ribosomal RNA (rRNA) gene. For the 28S rDNA PCRs, three primer sets were used (Table 2). For the assay using primers from Chenuil *et al.* [28], a 25-μL total reaction contained 1X GoTaq^®^ Flexi PCR Buffer (Promega, Madison, WI, USA), 0.4X Invitrogen Rediload™ loading buffer (Thermo Fisher Scientific, Waltham, MA, USA), 1.5 mM MgCl_2_, 0.4 mM dNTPs, each primer at 0.5 μM, 0.05 U μL^−1^ Promega GoTaq^®^ DNA polymerase, and 2.5 or 5 μL template DNA. Cycling was as follows: 95 °C for 5 min followed by 35 cycles of 95 °C for 30 s, 45 °C for 30 s, 72 °C for 1.5 min and then followed by 72 °C for 5 min. The 28S and 18S rDNA PCR assays using primers from García-Varela and Nadler [39] (Table 2) differed as 1 μM of each primer, 2 mM MgCl_2_, and 1 μL of template was used; cycling followed García-Varela and Nadler [39] with an annealing temperature of 56 °C. The ITS region rDNA PCR used the same reagents and concentrations as in the first PCR described above, and cycling was done as in Song *et al.* [93]. Amplification of partial digenean 28S and the second ITS region (ITS2) of the rRNA gene was done as in Hill-Spanik *et al.* [47].

**Table 1:** *Acanthogyrus* (*Acanthosentis*) species and sequences used in this study, including host(s), locality, GenBank accession numbers, and sequence lengths. If only one sequence length is listed for multiple accession numbers, the sequences are the same number of base pairs; otherwise, ranges are reported. Accession numbers in bold are from this study. There are mitochondrial cytochrome *c* oxidase I (mtCOI) sequences in GenBank for *Acanthogyrus cheni* (KX108947) and *A. kenyirensis* (MN833316), but our efforts to amplify mtCOI for *A.* (*A.*) cf. *tilapiae* were unsuccessful. *As of this submission, the species names were not updated in GenBank. 28S = large subunit ribosomal RNA (rRNA) gene, 18S = small subunit rRNA gene, ITS = the internal transcribed spacer region of the rRNA gene.

Species	Host(s)	Locality	18S		28S		ITS		
			Accession	Length (bp)	Accession	Length (bp)	Accession	Length (bp)	References
*Acanthogyrus* (*Acanthosentis*) *bilaspurensis*	*Cyprinus carpio*	Pakistan	OM262113, OM262040	1229	OM333893, OM333899	2805	–	–	[89]
*Acanthosentis cheni*	*Coilia nasus*	China	–	–	–	–	JX960708–JX960752	805–806	[93]
*Acanthogyrus* (*Acanthosentis*) *fusiformis*	*Arius* sp.	Vietnam	MK834518, MK834520	1710–1735	–	–	MK834517, MK834519	750–758	[11]
*Acanthogyrus* (*Acanthosentis*) *kashmirensis*	*Schizothorax plagiostomus*	India	MW000900, MW042815,MW042816	1295	–	–	MW000899, MW042813,MW042814	725	[91]
*Acanthogyrus* (*Acanthosentis*) *maroccanus*	*Luciobarbus callensis*	Algeria	–	–	MK953673	1085	–	–	[70]
*Acanthogyrus* (*Acanthosentis*) kenyirensis*	*Barbonymus schwanefeldii*	Malaysia	–	–	–	–	MK069588	813	[73]
Acanthosentis seenghalae*	*Puntius sophore*	India	KY305529	913	–	–	–	–	[41]
*Acanthogyrus* (*Acanthosentis*) tembatensis*	*Barbonymus schwanefeldii*	Malaysia	–	–	–	–	MK184205	640	[73]
*Acanthogyrus* (*Acanthosentis*) terengganuensis*	*Barbonymus schwanefeldii*	Malaysia	–	–	–	–	MK184204	589	[73]
*Acanthosentis tilapiae*	Unknown	“Atlantic Ocean”	–	–	U53000	311	–	–	[28]
*Acanthogyrus* (*Acanthosentis*) cf. *tilapiae*	*Oreochromis aureus, Coptodon guineensis*	Morocco	**OP765564** – **OP765571**	1005–1703	**OP502080** **,** **OP498339** **–** **OP498345**	686–2649	**OP498327** **–** **OP498333**	746–775	This study
*Acanthogyrus* sp. NIE–20129	*Oreochromis niloticus*	Egypt	MN709045	859	–	–	–	–	Unpublished
*Acanthogyrus* sp. 1 NKG-2016	Unknown	Unknown	KY305529	913	–	–	–	–	Unpublished
*Acanthogyrus* sp. 2 NKG-2016	Unknown	Unknown	KY305530	911	–	–	–	–	Unpublished
*Acanthogyrus* sp. KR-2022 isolate MK2	Unknown	Unknown	OP541602	1610	–	–	–	–	Unpublished
*Acanthogyrus* sp. KR-2022 isolate MK3	Unknown	Unknown	OP541603	1550	–	–	–	–	Unpublished
*Acanthogyrus* sp. KR-2022 isolate MK5	Unknown	Unknown	–	–	OP476684	1533	–	–	Unpublished
*Acanthogyrus* sp. KR-2022 isolate MK6	Unknown	Unknown	–	–	OP476685	1636	–	–	Unpublished
*Acanthogyrus* sp. KR-2022 isolate MK7	Unknown	Unknown	–	–	OP476686	1619	–	–	Unpublished
*Acanthogyrus* sp. KR-2022 isolate MK8	Unknown	Unknown	–	–	OP476687	1515	–	–	Unpublished

**Table 2:** Primers used for PCR amplification and sequencing; primers used only for sequencing are indicated by an asterisk. 28S = a portion of the large subunit ribosomal RNA (rRNA) gene, 18S = a portion of the small subunit rRNA gene, ITS = the internal transcribed spacer region of the rRNA gene. For primer orientation, + = sense, − = antisense.

Parasite	Marker	Primer Name	Primer Orientation	Primer Sequence (5′–3′)	Reference
Acanthocephalan	28S	LSU amplicon 1 forward	+	CAAGTACCGTGAGGGAAAGTTGC	[39]
		LSU amplicon 2 reverse	–	CTTCTCCAACKTCAGTCTTCAA	[39]
		LSU amplicon 3 forward	+	CTAAGGAGTGTGTAACAACTCACC	[39]
		LSU amplicon 4 reverse	–	CTTCGCAATGATAGGAAGAGCC	[39]
		LSU amplicon 1 reverse*	+	CAGCTATCCTGAGGGAAAC	[39]
		LSU amplicon 2 forward*	–	ACCCGAAAGATGGTGAACTATG	[39]
		LSU amplicon 3 reverse*	+	AATGACGAGGCATTTGGCTACCTT	[39]
		LSU amplicon 4 forward*	–	GATCCGTAACTTCGGGAAAAGGAT	[39]
		c72	+	GTGCAGATCTTGGTGGTAGT	[28]
		c9	–	TACTTAAGAGAGTCATAGTT	[28]
	18S	SSU forward	+	AGATTAAGCCATGCATGCGT	[40]
		SSU reverse	–	GCAGGTTCACCTACGGAAA	[40]
		SSU internal forward*	+	AGACGAACAACTGCGAAAGC	This study
		SSU internal reverse*	–	AGTTGTTCGTCTTGCGGTGA	This study
	ITS	BD1	+	GTCGTAACAACGTTTCCGTA	[64]
		BD2	–	TATGCTTAARTTCAGCGGGT	[64]
Digenean	28S	LSU5	+	TAGGTCGACCCGCTGAAYTTAAGCA	[53]
		28S_ECD2	–	CTTGGTCCGTGTTTCAAGACGGG	[96]
	ITS2	GA1	+	AGAACATCGACATCTTGAAC	[13]
		ITS2-2	–	CCTGGTTAGTTTCTTTTCCTCCGC	[30]

Products were electrophoresed on 1% agarose gels stained with GelRed (Biotium, Fremont, CA, USA) and visualized under a UV light. Samples that did not produce a band, or produced a faint band, were subjected to another round of PCR, which was done as above except the template was the product from the first PCR (instead of genomic DNA). Products were cleaned using ExoSAP-IT (Affymetrix, Santa Clara, CA, USA) and sent to Eurofins MWG Operon LLC (Louisville, KY, USA) for direct, bi-directional sequencing. All PCR and sequencing primers are listed in Table 2. For the 18S rRNA gene region, we designed two internal sequencing primers using Primer-BLAST [102] in order to generate bidirectional sequence for this marker (Table 2).

Complementary sequences were assembled, compared to their chromatograms, and edited accordingly using Sequencher version 5.4 (Gene Codes Corp., USA). Resulting sequences were compared to those in GenBank using BLASTN (Basic Local Alignment Search Tool [6]) and deposited in GenBank. All sequences from each marker in this study were then aligned with one another to examine any differences among specimens. The longest sequence for each respective marker was then aligned with sequences from GenBank (see Table 1 for acanthocephalans; see below for digeneans) except for the digenean ITS rDNA sequences for which the BLASTN queries resulted in an identical match to an existing GenBank sequence. ClustalW was used to generate acanthocephalan 18S rDNA and digenean 28S rDNA sequence alignments in MEGA11 [95]. NGPhylogeny.fr webservice [61] was used to implement MAFFT [54] for acanthocephalan 28S and ITS rRNA gene sequence alignments. Multiple alignments of the 28S rDNA data were generated in order to include as many nucleotides as possible in *p*-distance calculations given the high variation in length of GenBank sequences. NGPhylogeny.fr webservice [61] was also used to implement Gblocks [27] for selection of conserved regions of the resulting acanthocephalan ITS rRNA gene sequence alignment for use in subsequent *p*-distance calculations. All alignments were trimmed to remove any gaps on the terminal ends, and *p*-distances were calculated in MEGA11 [95].

### Population dynamics and statistics

Definitions (prevalence, intensity, etc.) follow Bush *et al.* [26]. Sex-ratio (male: female) was determined for each season by calculating the total number of female worms over total number of male worms. Descriptive statistics and regressions were calculated using Excel. Seasonal prevalences were compared via χ^2^ tests. Significance of regression results were determined using Spearman’s rho (*r*_s_; two-tailed). Seasonal differences in intensity, the effect of sex on intensity, and the effect of fish size on abundance were determined using Kruskal–Wallis tests (https://www.socscistatistics.com/). To assess if transmission of acanthocephalans peaked at different seasons, we analyzed female worm trunk length (*L*) as a proxy for worm maturity. Because of unequal sample variances in these latter data, we used Welch’s ANOVA (Minitab) and Welch’s *t*-tests (Excel) to determine significant differences. Mean intensities and mean lengths are expressed as means ± standard error. Results were considered significant at *P* ≤ 0.05.

## Results

**Figure 2:**
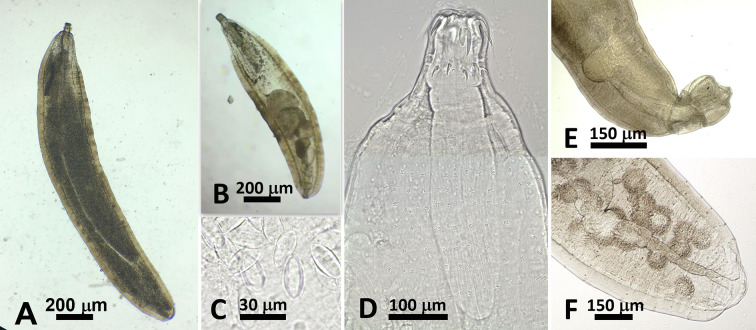
Acanthocephalan *Acanthogyrus* (*Acanthosentis*) cf. *tilapiae* from *Coptodon guineensis* at Sebkha Imlili. Fresh preparations. A. Female worm (gravid). B. Male worm. C. Ellipsoid eggs in gravid female. D. Proboscis and anterior trunk (montage) of male worm. Note strong anterior hooks and abruptly smaller middle and posterior hooks as well as regular rows of spines that were lost and leave rosette marks on tegument. E. Posterior end of male worm showing terminal genital opening and everted copulatory bursa. F. Posterior end of female showing terminal genital opening.

**Figure 3:**
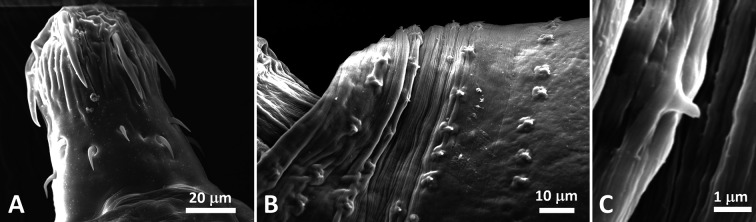
Acanthocephalan *Acanthogyrus* (*Acanthosentis*) cf. *tilapiae* from *Coptodon guineensis* at Sebkha Imlili. SEM. A. Male proboscis showing large anterior hooks markedly separated from small posterior hooks. B. Anterior trunk of female showing rows of spines. C. Female body spine.

**Figure 4:**
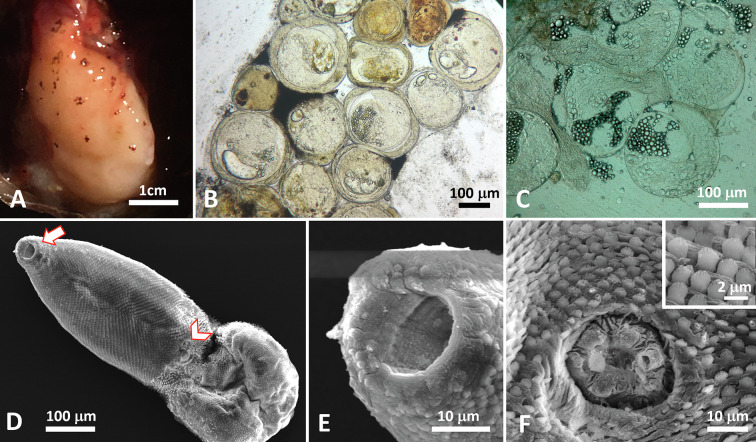
Metacercariae of heterophyid *Pygidiopsis genata* from *Coptodon guineensis* at Sebkha Imlili. A. Numerous metacercariae encysted on the outer wall of stomach. B & C. Fresh squashes of infected tissues with clusters of live metacercariae. D. SEM of excysted metacercariae showing a pyriform scaled body with terminal oral sucker (arrow) and subequatorial acetabulum (arrowhead). E. Oral sucker unarmed. F. small ventral sucker. Insert: pectinate body scales.

**Figure 5:**
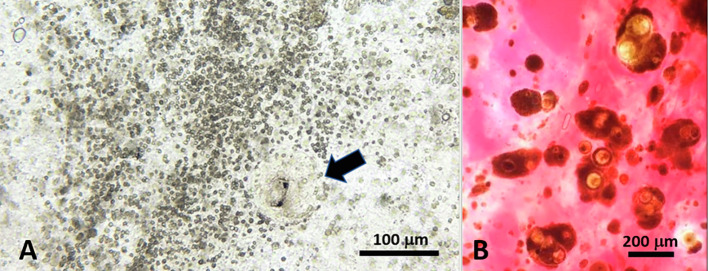
Unidentified metacercariae from *Coptodon guineensis* at Sebkha Imlili. A. Metacercaria (arrow) showing ocelli and associated with intense granulocytic reaction in intestinal mucosa. B. Fresh squash of spleen showing numerous metacercariae.

Individuals of *C. guineensis* were infected in their intestines by adult acanthocephalans (Figs. 2 and 3) and in various organs (kidney, spleen, gastric wall) by two types of metacercariae (Figs. 4 and 5). No monogenean was found on gills (*N* = 132). Eight out of the 12 specimens of *O. aureus* were infected by acanthocephalans in their intestines and by one type of metacercariae in their mesenteries; the two individuals of *C. zillii* were uninfected.

### Morphological identification of the acanthocephalan (Figs. 2 and 3; Tables 3 and 4)

Host: *Coptodon guineensis* (Günther, 1862)

**Table 3:** Measurements of main morphological characters of specimens of *Acanthogyrus* (*Acanthosentis*) *tilapiae* described by Baylis, 1947 [19] and redescribed by Troncy, 1970 [97] and Amin, 1978 [7], *A.* (*A.*) *papilio* described by Troncy and Vassiliadès, 1974 [97], and specimens (all stages of maturity combined) from this study collected from *Coptodon guineensis* at Sebkha Imlili. *n* = number of specimens studied. na = no data available. Merged data indicate no precision for male or female. *L* = length. *W* = width. All measurements are in μm unless indicated otherwise.

		*A.* (*A.*) *tilapiae*			*A.* (*A.*) cf. *tilapiae*	*A.* (*A.*) *papilio*
Reference		Baylis, 1947 [19]	Troncy, 1970 [97]	Amin, 1978 [7]	This study	Troncy & Vassiliadès, 1974 [99]
*n*		~65	18 males	27 males	50 males	2 males
			15 females (dead-suspect pseudoparasitism)	52 females	63 females	2 immature females
Trunk (mm) *L* × *W*	Male	2.5–3.5 × 0.8–1.1	Largest = 1.53	1.2–3.4 × 0.4–0.84	0.4–2.7 × 0.1 × 0.6	1 × 0.35
	Female	3–8 × 0.38–1.88	Largest = 2	1.2–5 × 0.32–1.32	0.3–5.8 × 0.1–0.9	1.025 × 0.27 (*n* = 1 immature)
Proboscis *L* × *W*	Male	na	65–110 × 60–100	90 × 64	60–400 × 40–400	na
	Female	110 × 100 (*n* = 2)		93–106 × 86–96	30–100 × 70 × 100	
Hook ant. Blade/root	Male	na	36–48/25–30	45	27–43/17–30	35–40/20–22
	Female	46–48		48–58	26–52/17–39	
Hook middle Blade/root	Male	na	12–20/20	16	10–17	8–11/5–6
	Female	12		13–22	10–17	
Hook post. Blade/root	Male	na	10–18/12	13	11–18/10–18	8–11/5–6
	Female	10		13–16	10–21/8–16	
Testis ant. *L* × *W*		Large	230–260 × 220–250	224–770 × 112–616	19–492 × 20–295	~170 × 170
Testis post. *L* × *W*				224–700	33–485 × 16–280	~200 × 200
Säefftigen’s pouch *L* × *W*			na	70–616 (L)	150–250 × 50–75	150 × 40
Cement gland			140–200		140–210 × 100–150	130 × 90
Egg		26–28 × 11	30 × 12	16–22 × 6–10	22–36 × 10–21	na
Body spines	Male	32–34	na	28–38	33–40	na
Number of rows	Female			28–42	25–40	
Body spines *L*		Minute	5		1–2	1–2

**Table 4:** Morpho-anatomical metrical data for female specimens (immature with or without ovarian balls, and gravid) and male specimens (all stages of development combined) of *Acanthogyrus* (*Acanthosentis*) cf. *tilapiae* from *Coptodon guineensis* at Sebkha Imlili. Data are averages in μm, followed by ranges in parentheses and number of specimens studied in italics. *L* = length; *W* = width; na = data not available.

		Immature females	Gravid females	Males
Proboscis	*L*	76 (28–115) *11*	86 (56–105) *12*	97 (59–414) *20*
	*W*	70 (53–105) *11*	71 (54–95) *12*	79 (41–362) *20*
Neck	*L*	66 (21–94) *10*	84 (60–117) *10*	83 (53–177) *12*
	*W*	41 (21–83) *10*	29 (16–48) *10*	28 (14–74) *12*
Trunk	*L*	1202 (348–1990) *26*	3411 (2014–5755) *27*	1298 (388–2715) *50*
	*W*	308 (103–522) *25*	599 (307–873) *27*	345 (117–647) *50*
Receptacle	*L*	144 (53–227) *11*	231 (150–328) *24*	148 (66–411) *22*
	*W*	48 (24–64) *11*	76 (51–108) *24*	65 (29–237) *22*
Lemniscus 1	*L*	464 (268–720) *14*	796 (346–1461) *13*	427 (270–880) *25*
	*W*	29 (10–52) *15*	60 (37–101) *13*	35 (14–57) *28*
Lemniscus 2	*L*	383 (156–516) *6*	716 (399–1049) *13*	533 (358–840) *8*
	*W*	32 (10–51) *5*	51 (30–79) *12*	40 (15–84) *34*
Hook I (ant.)	blade	40 (32–50) *16*	38 (26–52) *17*	36 (27–43) *29*
	root	26 (19–39) *16*	25 (17–36) *17*	24 (17–30) *29*
Hook II (med.)	blade	12 (10–12) *3*	15 (12–17) *9*	13 (10–17) *13*
	root	na	na	na
Hook III (post.)	blade	15 (11–21) *8*	13 (10–17) *13*	13 (11–18) *17*
	root	11 (10–13) *6*	12 (8–16) *12*	14 (10–18) *15*
Trunk spines	# rows	38 (33–43) *8*	35 (27–45) *9*	34 (25–40) *10*
Genital/trunk ratio				648 (178–1730) *36*
Eggs	*L*		31 (22–36) *22*	
	*W*		14 (10–21) *14*	
Testis (ant.)	*L*			220 (19–492) *36*
	*W*			158 (20–295) *28*
Testis (post.)	*L*			219 (33–485) *30*
	*W*			181 (16–280) *31*
Cement gland	*L*			174 (140–210) *5*
	*W*			125 (100–150) *5*
Säefftigen’s pouch	*L*			220 (150–250) *5*
	*W*			67 (50–75) *5*
Everted bursa	*L*			148 (100–250) *5*
	*W*			118 (100–180) *5*

Site of infection: small intestine (posterior to stomach)

Localities: Sebkha Imlili, Morocco (23°16′35.21″ N, 15°54′55.47″ W; 23°16′21.35″ N, 15°55′17.42″ W); Oued Aabar (27°56′09.9″ N, 11°25′24.1″ W)

Dates of collection: December 2018 and April, July, and October 2019

Other hosts and localities: *Oreochromis aureus* (Steindachner, 1864) at Guelta Kehla, Drâa Valley (28°26′60″ N, 10°51′35.999″ W) and Guelta Mrimima, Drâa Valley, Morocco (29°47′03.6″ N, 07°10′20.1″ W)

Date of collection: July 2019

Vouchers deposited: MNHN HEL1881 – HEL1888

GenBank accession numbers: 28S rDNA from host *C. guineensis*: OP498339–OP498341, OP498344–OP498245 / *O. aureus*: OP498343, OP502080. 18S rDNA from *C. guineensis*: OP765564–OP765567, OP765570–OP765571 / *O. aureus*: OP765568–OP765569. ITS region rDNA from *C. guineensis* OP498327–OP498329, OP498332–OP498333 / *O. aureus*: OP498330–OP498331.

Eoacanthocephala Van Cleave, 1936, Quadrigyridae Van Cleave, 1920, Pallisentinae Van Cleave, 1928, with characters of the genus *Acanthogyrus* Thapar, 1927 and subgenus *Acanthosentis* Verma and Datta 1929: males and females small with body showing a ventral curvature and covered with minute spines (1–2.5 long) in 36 (25–45) complete rows closer to one another on ~anterior third of body (~level of lemnisci) and more spaced out in middle of body. Proboscis cylindrical, small, armed with 6 spirals of 3 hooks (18 total). No apical organ observed. Anterior hooks markedly separated and larger than middle and posterior hooks. Hook roots simple, shorter than blades. Proboscis receptacle single-walled with ganglion at its base. Lemnisci elongate, >3× longer than receptacle, one slightly longer than the other (considered subequal). Genital opening terminal in both sexes.

Males: Based on 50 specimens in wet mount, 8 in Canada balsam or Kleermount, and 1 for SEM. Trunk 1298 (388–2715) × 345 (117–647) *n* = 50. Proboscis 97 (59–414) × 79 (41–362) *n* = 20. Number of rows of spines 34 (25–40) *n* = 10). Anterior, middle, and posterior hooks 36 (27–43) *n* = 29), 13 (10–17) *n* = 13, 13 (11–18) *n* = 17, respectively. Roots of anterior and posterior hooks (middle hook roots not measured) 24 (17–30) *n* = 29, 14 (10–18) *n* = 15, respectively. Neck 83 (53–177) × 28 (14–74) *n* = 12. Proboscis receptacle 148 (66–411) × 65 (29–237) *n* = 22 wide. Lemnisci subequal 533 (358–840) *n* = 25 × 35 (14–57) *n* = 28. Reproductive system occupying approximately 50% (46–64%) of trunk length *n* = 36. Testes equatorial, ovoid, in tandem, often slightly overlapped. Anterior testis 220 (19–492) *n* = 36 × 158 (20–295) *n* = 28. Posterior testis 219 (33–485) *n* = 30 × 181 (16–280) *n* = 31. Vas deferens swollen to form seminal reservoir. Cement gland subspherical, 174 (140–210) *n* = 5 × 125 (100–150) *n* = 5, with 5 giant nuclei *n* = 1. Saefftigen’s pouch 220 (150–250) × 67 (50–75) *n* = 5. Everted copulatory bursa 148 (100–250) × 118 (100–180) *n* = 5.

Females: Based on 63 specimens, 28 immature or with ovarian balls (26 in wet mount and 2 in Kleermount) and 35 gravid (27 in wet mount, 7 in Canada balsam or Kleermount and 1 for SEM). Trunk 2306 (348–5755) × 448 (103–873) *n* = 54. Proboscis 81 (28–115) × 71 (53–105) *n* = 23. Anterior, middle and posterior hooks 36 (26–52) *n* = 35, 13 (10–17) *n* = 12, 14 (10–21) *n* = 21 long, respectively. Roots of anterior and posterior hooks (middle not measured) 25 (17–39) *n* = 33, 11 (8–16) *n* = 18 long, respectively. Neck 75 (21–117) 20 × 35 (16–83) *n* = 20. Proboscis receptacle 187 (53–328) × 62 (24–108) *n* = 35. Lemnisci subequal 630 (268–1461) × 44 (17–39) *n* = 33. Mature eggs ellipsoid 31 (22–36) *n* = 22 × 14 (10–21) *n* = 14.

### Molecular identification of the acanthocephalan

Partial 28S rRNA gene sequences (*n* = 6 from *C. guineensis*, *n* = 2 from *O. aureus*; 686–2649 bp) from the acanthocephalan were 99.9% similar to one another where there was overlap, and 92–96% similar (98% BLAST query coverage) to sequences from *A.* (*A.*) *bilaspurensis* Chowhan *et al.* 1987 [29] collected from carp *Cyprinus carpio* in Pakistan (OM333893, OM333899). The *A*. (*A*.) *maroccanus* Dollfus, 1951 [33] sequence collected from a specimen found in barb *Luciobarbus callensis* from Algeria (MK953673 [70]) only encompasses the D1–D3 regions of the 28S rRNA gene and differed by 16% based on a 757-bp alignment. The 28S rDNA sequence in GenBank of *A.* (*A.*) *tilapiae* collected from an unknown host in the “Atlantic Ocean” (U53000) is very short (311 bp containing the D7 region; [28]) and was only 0.64% (or 2 bp) different from our sequences, while *A. bilaspurensis* (the only other named species with D7 region 28S rDNA sequencing data in GenBank) differed from our sequences and *A. tilapiae* by 2.6% (327-bp alignment). The other D7 region 28S rDNA GenBank sequences for *Acanthogyrus* spp. in GenBank are unpublished (OP476684–OP476687), and *p*-distances ranged from 6.3% to 11.1% based on the 327-bp alignment (and 6.7–11.0% based on a 1630-bp alignment).

Partial 18S rRNA gene sequences (*n* = 6 from *C. guineensis*, *n* = 2 from *O. aureus*; 1005–1703 bp) were 100% similar to one another and were again most similar to *A.* (*A.*) *bilaspurensis* sequences (98.3% similarity to OM262113, OM262040 with 70% BLAST query coverage). ITS rRNA gene sequences (*n* = 5 from *C. guineensis*, *n* = 2 from *O. aureus*; 746–775 bp) were 99.9% similar to one another and 91% similar to sequence from *A.* (*A.*) *terengganuensis* Mohd-Agos *et al.*, 2021 [73] collected from tinfoil barb *Barbonymus schwanefeldii* in Malaysia (MK184204) with very low BLAST query coverage (24–27%) due to the very few numbers of conserved positions; GBlocks only detected 380 conserved positions (out of 934 bp) in the ITS rRNA gene sequence alignment. Across the 380 bp, *p*-distances ranged from 33.9% (*A. kashmirensis* Amin *et al.*, 2017 [12]; MW000899, MW042813–MW042814) to 45.7% (*A. tembatensis* Mohd-Agos *et al*., 2021 [73]; MK184205).

#### Remarks

Five species of *Acanthogyrus* (*Acanthosentis*) species are known from Africa. *Acanthogyrus* (*A.*) *maroccanus*, is known from several barb fish species and is considered endemic in North Africa [33, 68, 70]. Specimens of this species have significantly larger anterior, middle, and posterior proboscis hooks (62 μm, 62 μm, 48 μm, respectively) and only 12–18 rows of spines on the body (see redescription [70]), and our 28S rRNA gene sequences differed from the *A*. (*A*.) *maroccanus* sequence by 16%. Specimens of *A*. (*A*.) *nigeriensis* Dollfus and Golvan, 1956 [34] have significantly larger bodies, proboscis hooks, and body spines size and circle numbers. Specimens of *A*. (*A*.) *malawiensis* Amin and Hendrix, 1999 [10] have proboscis hooks from the medial circle larger than the anterior hooks and larger body spines. The species *A.* (*A.*) *papilio* Troncy and Vassiliadès, 1974 [99] was described from the mudskipper *Periophthalmus papilio* Bloch-Schneider (now *P. barbus* L.) based on the very small size of the specimens and anterior proboscis hooks that were markedly larger (35–40 μm) than the medial and posterior hooks (8–11 μm long). Body spines are minute and totally cover the trunk of the specimens. It is important to note that the very brief description of *A. papilio* is based on four specimens in poor condition according to the authors: two males (no maturity stage given) and two immature females, and that the sizes of our immature specimens as well as their proboscis hooks encompass the sizes provided in the description of *A. papilio* (Table 3); it is possible that the mudskipper could be an accidental host preventing full development of the worms. However, the types, although listed in the original manuscript as having been deposited at the Museum of Natural History in Paris, France, are non-existent and our efforts to obtain acanthocephalan specimens from this area were fruitless. Further, there are no sequencing data for *A*. (*A*.) *nigeriensis*, *A*. (*A*.) *malawiensis*, or *A. papilio* in GenBank. The fifth species, *A*. (*A.*) *tilapiae* [19], has a broad distribution throughout continental Africa and Madagascar and is reported from over 10 species of tilapia (e.g., [7, 10, 66, 68]) but also in the pufferfish *Tetraodon fahaka* (see [98] in [10]) and bagrid *Labeo cylindricus* [10]. Recent studies using SEM have brought up new morphological details [9, 68], in particular regarding body spine distribution, size, and shape. Given our data and the very little information available on *A. papilio* compared to *A.* (*A.*) *tilapiae*, we conclude that our specimens most resemble *A.* (*A.*) *tilapiae* by their size and distribution and size of their proboscis hooks and body spines. Also, while the 28S rDNA sequence in GenBank of *A.* (*A.*) *tilapiae* is only 311 bp and the origin of the specimen from which it was generated may be questionable (see Discussion), it was only 0.64% different from that of our specimens, while *A. bilaspurensis* differed from our sequences and *A. tilapiae* by 2.6%. Therefore, based on the information available at this time and because of a possible taxonomic issue with *A. papilio*, we identify the specimens we collected from individuals of *C. guineensis* and *O. aureus* as *A*. (*A.*) cf. *tilapiae.* Comparative measurements of the main morphological characters of *A. tilapiae*, *A. papilio,* and our specimens are in Table 3.

### Morphological and molecular identification of metacercariae (Fig. 4)

Host: *Coptodon guineensis* (Günther, 1862)

Sites of infection: surface of stomach and liver, kidney, ovaries, more rarely muscle

Locality: Sebkha Imlili, Morocco (23°16′35.21″ N, 15°54′55.47″ W; 23°16′21.35″ N, 15°55′17.42″ W)

Other hosts and localities: *Oreochromis aureus* (Steindachner, 1864) at Guelta Mrimima, Drâa Valley (MK955803), Morocco (29°47′03.6″ N, 07°10′20.1″ W)

Date of collection: July 2019

GenBank accession numbers: 28S rDNA from host *C. guineensis*: OP498346–OP498349 / *O. aureus*: OP498350–OP498351. ITS region rDNA from *C. guineensis*: OP498336–OP498338, OP481215 / *O. aureus*: OP498334–OP498335.

We were successful at excysting metacercariae and obtaining sequences for only one of the two types, which was found both in *C. guineensis* at Sebkha Imlili and *O. aureus* at one of the gueltas. Heterophyidae Leiper, 1909. Based on two excysted specimens for SEM: body pyriform 289 (278–300) *n* = 2 × 90 (89–91) *n* = 2 at ventral sucker level and 118 (118) *n* = 1 at widest (Fig. 4D). Posterior end wider than anterior end. Body covered with pectinate scales 2 (1.7–2.6) *n* = 11 (Fig. 4F, insert). Mouth subterminal; oral sucker unarmed 25 (24.8–26.3) *n* = 2 in diameter (Fig. 4E). Ventral sucker subequatorial, smaller than oral sucker, 16 (15–17) *n* = 2 in diameter. Given that this description is based on metacercariae, no attempt to compare with species descriptions was made and worms were identified molecularly. ITS rRNA gene sequences (*n* = 6, 353–369 bp long) were identical to one another and to that from a specimen of *Pygidiopsis genata* Looss, 1907 (AY245710) collected from cormorant *Phalacrocorax carbo* in Israel [36]. Our partial 28S rRNA gene sequences (*n* = 6, 862–877 bp) were identical to one another, and when aligned with 28S rRNA gene sequences from the only two species of *Pygidiopsis* in GenBank, *P. summa* Onji and Nishio, 1916 obtained from an experimental infection of an unknown host (AF181885) and *P. macrostomum* Travassos, 1928 from experimentally-infected hamster *Mesocricetus auratus* in Brazil (MF972527–MF972531, KT877409) and from greater bulldog bat *Noctilio leporinus* in Mexico (MW332629), *p*-distances were 9.5% (243-bp alignment) and 12.5% (816 bp-alignment), respectively.

### Unidentified metacercaria (Fig. 5)

Metacercariae of a presumed single but unidentified species were found only in *C. guineensis* at Sebkha Imlili. Specimens showed ocelli, measured ~70 μm diameter in fresh squashes, and were mainly encysted in the spleen, ovaries, kidney, and the intestinal mucosa of the fish where they were associated with intense granulocytic reaction (Figs. 5A–5B).

### Population dynamics of parasites in *C. guineensis*

Overall, 68.8% (221/322) of fish were infected by *A.* (*A.*) cf. *tilapiae* (mean intensity 4.6 ± 0.29), 74.15% (194/264) by metacercariae of *P. genata*, and 21.5% (50/232) by the unidentified metacercariae. For all three parasites, fish as small as 20 mm in TL were infected. Infection results are reported in Table 5.

**Table 5:** Prevalence (P), mean intensities (MI), and mean abundance (MA) of infection of *Coptodon guineensis* at Sebkha Imlili by acanthocephalan *Acanthogyrus* (*Acanthosentis*) cf. *tilapiae* and metacercariae (mc) of *Pygidiopsis genata* and of an unidentified (unid.) species. *N* = sample size; SE = standard error; TL = Total length of fish in mm.

Dates of collection	TL (range)	*Acanthogyrus* (*A*.) cf. *tilapiae*			mc *Pygidiopsis genata*	mc unid.
		P% (N)	MI ± SE (*N*)	MA ± SE (*N*)	P% (N)	P% (N)
Dec. 2018	89 ± 2.2 (17–139)	75.5 (93)	5.37 ± 0.5 (69)	3.89 ± 0.45 (93)	79.3 (93)	0 (93)
Apr. 2019	92.5 ± 2.7 (44–185)	50.6 (80)	2.44 ± 0.54 (41)	1.14 ± 0.30 (80)	56.3 (80)	0 (80)
Jul. 2019	92.4 ± 14.6 (49–166)	71.7 (92)	4.97 ± 0.53 (66)	3.43 ± 0.45 (92)	66.7 (39)	100 (25)
Oct. 2019	65 ± 4.3 (20–150)	78.9 (57)	4.86 ± 0.72 (45)	2.78 ± 0.57 (57)	94.3 (53)	71.4 (35)

**Figure 6:**
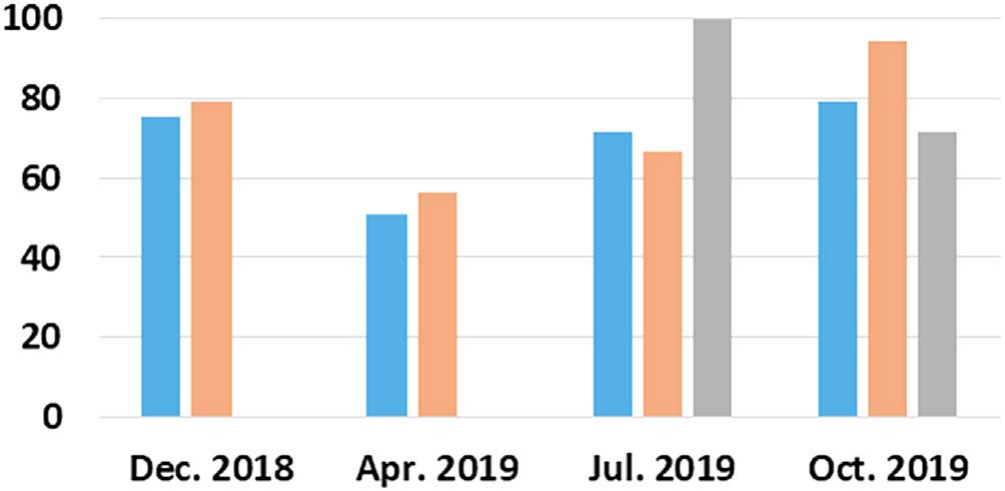
Prevalence of infection (%) of *Coptodon guineensis* at Sebkha Imlili. Blue bars = acanthocephalan *Acanthogyrus* (*Acanthosentis*) cf. *tilapiae* (December 2018: *n* = 93; April 2019: *n* = 80; July 2019: *n* = 92; October 2019: *n* = 57); Orange bars = metacercariae of *Pygidiopsis genata* (Dec 2018: *n* = 92; April 2019: *n* = 80; July 2019: *n* = 39; October 2019: *n* = 53); Grey bars = unidentified metacercariae (December 2018: *n* = 92; April 2019: *n* = 80; July 2019: *n* = 25; October 2019: *n* = 35).

**Figure 7:**
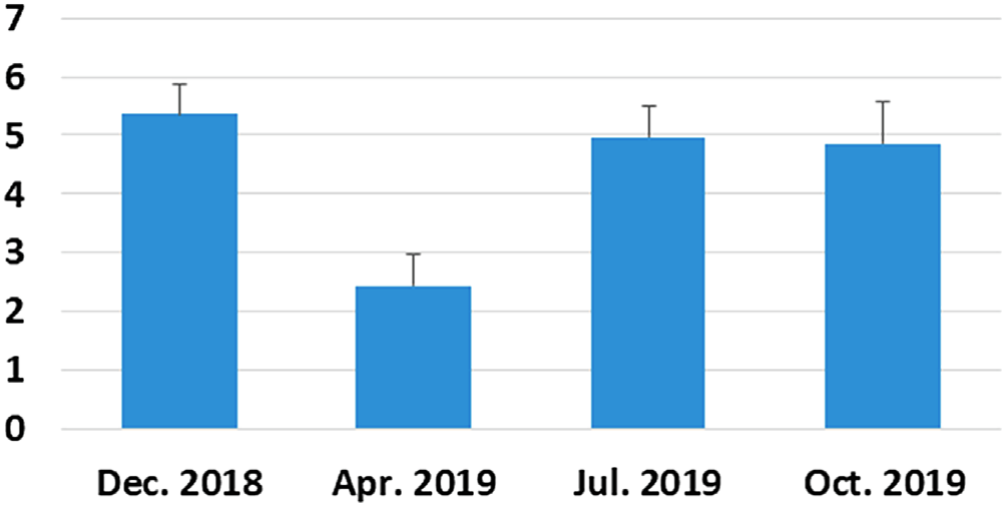
Mean intensity of infection of *Coptodon guineensis* at Sebkha Imlili by acanthocephalan *Acanthogyrus* (*Acanthosentis*) cf. *tilapiae* (December 2018: *n* = 69; April 2019: *n* = 41; July 2019: *n* = 66; October 2019: *n* = 45). Mean intensity was significantly lowest in April.

**Figure 8:**
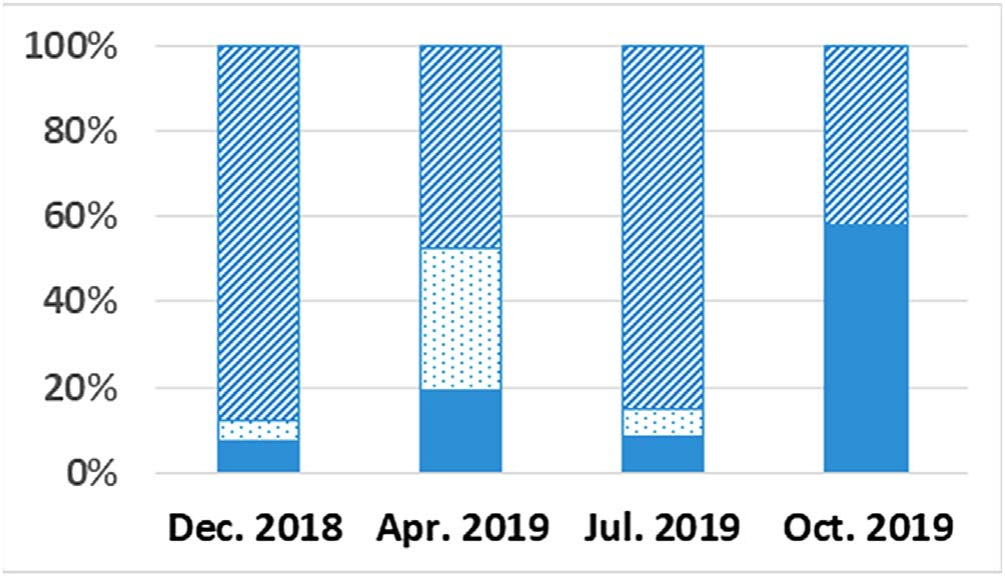
Proportions of females of acanthocephalan *Acanthogyrus* (*Acanthosentis*) cf. *tilapiae* in *Coptodon guineensis* at Sebkha Imlili according to stage of maturity. Solid bars = immature females (no genitalia visible); dotted bars = ovigerous females (ovarian balls visible); striped bars = gravid females (December 2018: *n* = 41; April 2019: *n* = 42; July 2019: *n* = 47; October 2019: *n* = 50). Gravid females were present throughout the year but significantly more abundant proportionally in December and July, which may indicate a short life span of the worms and quick turnover in the fish.

**Figure 9:**
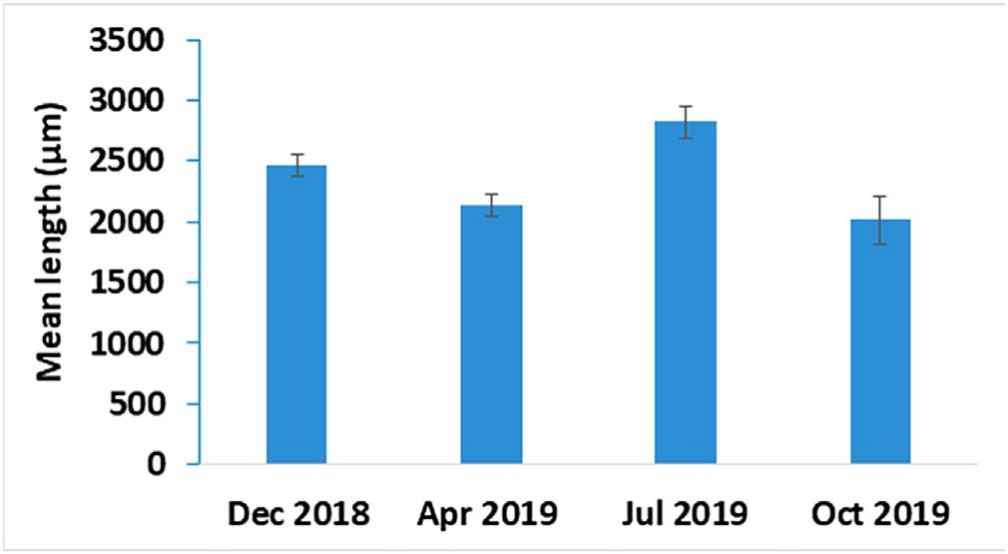
Mean trunk length of females of acanthocephalan *Acanthogyrus* (*Acanthosentis*) cf. *tilapiae* in *Coptodon guineensis* at Sebkha Imlili (December 2018: *n* = 41; April 2019: *n* = 42; July 2019: *n* = 47; October 2019: *n* = 50). Worms were significantly smaller in April and October compared to December and July.

**Figure 10:**
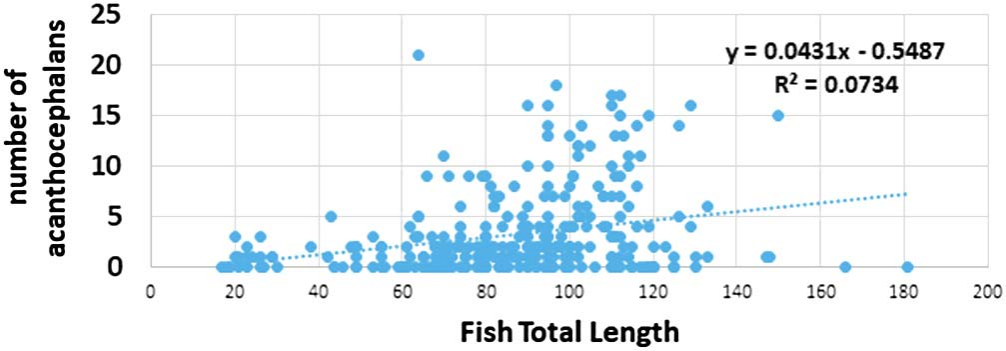
Abundance of infection of *Coptodon guineensis* at Sebkha Imlili by acanthocephalan *Acanthogyrus* (*Acanthosentis*) cf. *tilapiae* according to fish total length. Infection occurred in fish as small as 20 mm. Larger fish were more often infected than smaller fish.

The acanthocephalan was present throughout the four sampling periods, and there was no seasonal pattern of transmission with respect to prevalence (χ^2^ = 7.035, df = 3; *P* = 0.071) (Fig. 6). Mean intensity was significantly lower in April when ovigerous females were in highest proportion compared to the rest of the year (Kruskal–Wallis: *H* = 25.16; df = 3, *N* = 221, *P* < 0.01; Figs. 7 and 8). Gravid females were found throughout the year and in highest proportion in December and July (Fig. 8). Analysis of female acanthocephalans indicated a significant overall effect of season relative to female worm length (Welch’s ANOVA, *P* < 0.001; Fig. 9). Significantly longer (more mature) female worms were found during the summer (2820.5 ± 133.7) compared to the spring (2141.3 ± 89.4; *P* = 0.038) and in the winter (2460.2 ± 88.7) as compared to the fall (2013.4 ± 191.8; *P* < 0.001; Fig. 9). Sex-ratio was ~1 male: 2 females at each collection time (male to female ratios were 125:246, 31:69, 95:175, 78:137 for winter, spring, summer, and fall, respectively). There was no effect of fish sex on intensity of infection (Kruskal–Wallis: *H* = 3.66, df = 1, *N* = 152, *P* = 0.06 for males vs. females, and *H* = 3.82, df = 2; *n* = 210, *P* = 0.148 with inclusion of a group for undetermined sex). Fish size had no effect on prevalence of infection, but abundance (Spearman’s Rho: *r*_s_ = 0.263, *P* < 0.001, Fig. 10) and intensity (Spearman’s Rho: *r*_s_ = 1, *P* < 0.001) of infection increased with fish total length.

There was a significant seasonal pattern of transmission as prevalence of metacercariae of *P. genata* was significantly lower in April (56.2%) and highest in October (94.3%) (χ^2^ = 10.93, df = 3; *P* = 0.012) (Fig. 6). The unidentified metacercariae were encountered only in July and October when, overall, 85.7% of the 60 fish examined were infected (χ^2^ = 180.95, df = 3; *P* < 0.0001). Fish size had no effect on prevalence of infection of either metacercariae.

## Discussion

A large proportion of individuals of *C. guineensis* at Sebkha Imlili were co-infected throughout the year by adult eoacanthocephalans, *Acanthogyrus* (*Acanthosentis*) cf. *tilapiae*, in their intestines and metacercariae of heterophyid *P. genata* in various organs. Metacercariae of another unidentified digenean, encysted particularly in the spleen, were also present in July and October. Because the life cycle of this acanthocephalan is strictly aquatic, and because the fish have been isolated in the water holes since the Holocene [37], the occurrence of the acanthocephalan in definitive host *C. guineensis* indicates that the parasite infected this species when the current Sebkha was a lagoon, prior to marine regression and desertification [37]. Fish *C. guineensis* serve as second intermediate hosts for the digeneans, which use a gastropod as first intermediate host and terrestrial animal(s) as definitive host(s). The digeneans could be relics of the past but could also be more recently introduced via terrestrial vertebrates (definitive hosts) that use the Sebkha as a source for food or water (e.g., birds or mammals [83, 86]). In both cases, infections of the fish at the Sebkha occur due to the capacity of invertebrate intermediate hosts and free-living stages of the parasites to survive the extremely high salinities of the pools.

Our knowledge of digeneans that infect the fish at Sebkha Imlili is very limited. Given the significant seasonal pattern of prevalence of both metacercariae, it is possible that their definitive hosts are migratory. The presence of the unidentified metacercariae for only part of the year could also indicate the possible removal of infected fish from the population due to severe pathogenicity associated with their very high abundance in the spleen [79]. For *P. genata*, while adults can infect mammals and be zoonotic [65], the most common definitive hosts are piscivorous birds (e.g., pelicans and cormorants [37]), and metacercariae have been reported in several fish species including the freshwater tilapia, *C. zillii* [35, 52]. At the Sebkha, the predominant migratory birds (e.g., accipitrids and passerines) are not piscivorous and some have been reported to not survive in high salinity environments [81]. However, the cormorant *Phalacrocorax carbo marrocanus* and several ardeids (common hosts for other heterophyids [31, 59]) do frequent the Sebkha at various times of the year [81, 86] and are good candidate definitive hosts for *P. genata*. For both digeneans, the presence of metacercariae in the fish indicates that their life cycles certainly involve the only snail found in the water holes, the hydrobiid *Ecrobia ventrosa* (Montagu, 1803). This snail is broadly distributed in brackish areas and salt marshes along the Atlantic coast of Africa [42], although it is not known if it also inhabits the Oued Mrimima where we found individuals of *O. aureus* infected by metacercariae of *P. genata* as well. However, given that a known intermediate host for *P. genata* is the freshwater melanopsid, *Melanopsis costata* (Olivier, 1804) [35, 36], it is more likely that the specificity of this digenean for its gastropod first intermediate host is as broad as for its second intermediate and definitive hosts.

Regarding the acanthocephalan, *Acanthogyrus* (*A*.) *tilapiae* is reported from numerous cichlids of various genera in several localities throughout Africa (see checklist [56] and review [10]). Included in this broad host distribution is the redbelly tilapia *C. zillii* and the blue tilapia *O. aureus*, the latter of which we also found infected in the freshwater Oued Mrimima north of Imlili in Morocco, thus extending the geographical distribution of this parasite in Africa. While such a broad host and geographical distribution may hide a complex of species, which would result in the specificity for their definitive hosts being narrower than apparent, it is also possible that *A.* (*A*.) *tilapiae* is stenoxenous and owes its success to its flexibility for its definitive hosts (and possibly its intermediate hosts as well). It is, however, reportedly a freshwater parasite, as all known hosts to date inhabit lakes and rivers (e.g., [10, 19, 43, 44, 56]). Hence, its presence in hypersaline waters at Sebkha Imlili would have to be explained by it having switched to *C. guineensis* as definitive host from a freshwater tilapia (e.g., native *C. zillii* when the Sebkha was a lagoon fed by freshwater during the Holocene (Green Sahara period) [37]. In support of such a freshwater origin are that i) *C. guineensis* is known to occur in some freshwater rivers [2]; ii) the freshwater tilapia *O. aureus* and C*. zillii* (often reported as host for *A.* (*A.*) *tilapiae* elsewhere in Africa [15]) occur in Morocco in sympatry with *C. guineensis* [101]; and iii) the acanthocephalan we found in *C. guineensis* (both from Oued Aabar and Sebkha Imlili) as well as in *O. aureus* is the same species based on morphology and sequencing data. Such host shifting allows for host range expansion [14, 17] with multiple examples of such parasite host switching [63, 78]. Although parasite host switching might be easier among closely related hosts, it is not limited to closely related host species [25, 45], and it is typically the consequence of environmental changes that increase encounter opportunities (e.g., captivity or introduction of new species in an ecosystem [67]). However, successful switching of parasites with complex life cycles from freshwater to marine hosts also requires that both the parasite and the host(s) have the physiological capacity to survive altered conditions [3]; this translates as both a lack of specificity of the parasites for their intermediate host(s) and the capacity of both the free-living stages (i.e., eggs and larvae) and the intermediate hosts to withstand changes in salinity. Nothing is known about the life cycle of *A.* (*A*.) *tilapiae*; however, eggs (the only acanthocephalan free-living stage) and cystacanths (being provided ‘environmental stability’ inside their hosts) are resting stages that allow the parasite to survive otherwise potentially unfavorable environmental conditions [55]. Significantly, the potential intermediate hosts of the acanthocephalan from this study are limited to the only two microcrustaceans present in this simplified ecosystem, the ostracod *Cyprideis torosa* (Jones, 1850) (see [48]) and the harpacticoid copepod *Cletocamptus retrogressus* Schmankevitsch, 1875 (identified by Marc Pagano, IRD, Université de Marseille, pers. comm.). Both species are common in various localities in Morocco [18, 21, 75, 87] and these microcrustaceans are capable of withstanding substantial fluctuations in salinities, including survival in extreme salinities [48, 72]. Although both ostracods and copepods are the typical intermediate hosts of eoacanthocephalans [90], the two species of *Acanthosentis* whose life cycles are known use copepods [50, 92]. Of particular interest is the marine species, *A. lizae* Orecchia, Paggi and Radujkovic, 1988*,* reported, to our knowledge, as being the only acanthocephalan that uses a marine copepod in its life cycle, which also happens to be a harpacticoid copepod [50]. Hence, *C. retrograssus* is a very plausible intermediate host candidate for the acanthocephalan in the Sebkha. Consequently, while it is argued that it can be more difficult for parasites with complex life cycles to transition to new environments [5, 16, 20], at Sebkha Imlili all the hosts of this acanthocephalan are present in the water holes because the cycle is strictly aquatic, and they are all euryhaline capable, thus allowing the potential for the otherwise assumed freshwater *A.* (*A*.) *tilapiae* to have established itself successfully in this environment of extreme salinities.

While we conclude that *A.* (*A.*) *tilapiae* was the reasonable identification of our specimens given our current state of knowledge, the alternate identification as *A. papilio* would generate a very different scenario, which must be discussed herein because of its biological relevance. *Coptodon guineensis* is a coastal cichlid on the African west coast that ranges from Angola to the north of Morocco [4, 80, 82, 94]. Because *A. papilio* was described from mudskippers from mangroves in Senegal [99] where *C. guineensis* also lives [57], and because this genus of acanthocephalan is common in tilapia species in Africa, it is not unreasonable to also consider that *C. guineensis* could have been infected when the Sebkha was a lagoon [1, 37]. It is also plausible for such a marine parasite to have persisted over a long period of time in an environment where its intermediate hosts would already be present, as all organisms associated with the Sebkha are coastal or estuarine and known from other areas in Morocco [48]. To add to the argument, there is no host detail for the sole 28S rDNA sequence of *A.* (*A.*) *tilapiae* available in GenBank [28] other than the notation that it came “from the Atlantic Ocean and [was] provided by Dr. Mattei” (p. 579–580), who was a researcher in Dakar, Senegal (https://en.wikipedia.org/wiki/Xavier_Mattei#Career). Hence, it is likely that this specimen, identified as *A.* (*A.*) *tilapiae* [13], was from the Dakar area – the area where *A. papilio* was described (Joal-Fadiouth, Senegal [99]) and where *C. guineensis* also occurs [57]. In short, it is likely that the short GenBank sequence could as well have been from specimens of *A. papilio*. The 99.9–100% similarities among sequences (using all three markers) that we generated from acanthocephalan specimens taken from *C. guineensis* and *O. aureus* would imply that *A. papilio* is found in both marine and freshwater systems. This conundrum can be reconciled if we question the validity of *A. papilio*, as its description is based only on immature specimens and overlaps somewhat with that of *A.* (*A.*) *tilapiae.* We should also consider that the acanthocephalan species could be an altogether different and cryptic species because it has been isolated for a long period of time, and given that the *Coptodon* from the Sebkha Imlili is possibly an “incipient” species of tilapia [2]. Given what we know at this stage however, it is most reasonable to identify this acanthocephalan as *A.* (*A.*) cf. *tilapiae*. Future studies will need to investigate the validity of *A. papilio* as well as the genetics of *A.* (*A.*) *tilapiae* to disentangle the conundrum and identify a putative complex of species.

Unraveling the population dynamics of this acanthocephalan can help us understand how the Sebkha ecosystem functions and how it has persisted for such a long period of time. Both the abundance and intensity of infection by the acanthocephalan increased with total fish length. This is not unusual and is typically explained for trophically transmitted parasites by an increase or a shift in the diet of their hosts, sometimes amplified with the occurrence of paratenic hosts [29, 76] and/or an extended longevity of the adult worms [55]. This latter explanation, however, is unlikely for fish acanthocephalans that rarely live longer than a few months [55], which seems the case for the acanthocephalan we found. The simplest explanation is that larger individuals of *C. guineensis* graze and ingest more organisms at the bottom of the water holes (including copepods and ostracods [62]) than smaller fish; Significantly, larger fish also cannibalize smaller ones (pers. obs.). Snails and shrimp are the only organisms that could, in theory, fulfill the role of paratenic hosts for this parasite in this particular habitat, as they too graze at the bottom of the water holes. Snails and shrimp do not appear to be part of the fish diet, however, and no cystacanth was found in the ~40 shrimp we examined, although it must be noted that these examinations were sporadic. Furthermore, acanthocephalans typically use vertebrates as paratenic hosts [76], with the rare exception of some *Neoechinorhynchus* species that use invertebrates [58, 60]. Hence, the occurrence of paratenic hosts in this acanthocephalan life cycle is not expected but cannot be totally discounted for lack of thorough investigation. In contrast, there is evidence of cannibalism by fish larger than 60 mm, which indicates the possibility for eupostcyclic transmission of this acanthocephalan [22, 76], a phenomenon that, at least in part, could explain the higher intensities we found in larger fish [23, 77].

The distribution of adult acanthocephalans was aggregated, and the mean intensity of infection was ~5 worms per fish, which is quite low for hosts whose environmental conditions can be compared to that of captive animals. The non-occurrence of super infection (maximum intensity = 21 worms in only one fish out of the 322 examined) indicates the likelihood of a negative feedback mechanism acting as a regulatory factor to limit infrapopulations, which can explain that this host/parasite system has persisted for a very long time. Given the constant occurrence of immature and gravid females and the likelihood of the continuous presence of infective cystacanths in the pools’ microcrustaceans throughout the year (development of cystacanths in those is ~15–30 days [90]), along with low mean acanthocephalan intensities beg the question regarding which factor(s) operate in this system to regulate infection; however, at this stage of knowledge of this fish/parasite system, we can only speculate in this regard. For instance, several experimental studies of fish acanthocephalans demonstrated density-dependent survival of cystacanths associated with limited resources, such as space [100], which could be the case herein as the worms seem to occupy a short post-gastric portion of the intestine and thus may have a constricted “zone of viability” [55]. A somewhat unique character of the acanthocephalan population in fish at the Sebkha is that gravid female worms, which occurred throughout the year, were in significantly high proportion twice a year (December and July), which could indicate the occurrence of two marked reproductive periods per year. Overall, the acanthocephalan population in *C. guineensis* appears to display a dynamic equilibrium between recruitment and loss of parasites, possibly because of a continuous turn-over of infrapopulations in the fish coupled with the constant availability of infective cystacanths. The lower intensity observed in April may be an instance where we captured a shift in generations, as we also saw more immature females in that same collection period. Typically, factors linked to acanthocephalan cycles (e.g., length of prepatent and patent periods) are associated with host movements and environmental conditions (including seasonality). None of these factors apply to the acanthocephalan in the Sebkha, however, as there seems to be no particular period of unfavorable conditions in the water holes: environmental conditions are fairly stable throughout the year and water temperatures do not fluctuate as in higher latitudes, so no host appears to “overwinter” per se, and none of the hosts migrate. In this regard, the water holes display similar conditions to captivity. While there appears to be a cyclic pattern in female worm maturation, there is no obvious pattern of seasonal transmission as prevalence of infection was similar throughout the year, the male/female sex ratio was constant, and gravid and immature and mature females were present at all periods of collection, albeit in different proportions. This type of pattern is not unique (e.g., [8, 74]) but is atypical for acanthocephalans, which most often display a yearly cycle closely associated with seasonal periodicity or biotic factors such as a qualitative or quantitative change in host diet [55, 76]. Hence, this particular host/acanthocephalan system may project the dynamic modifications that can occur in some other parasite cycles, which should be taken into consideration with respect to global climate change [24]. Lastly, while mathematical models tend to oversimplify complex systems, the ecosystem at Sebkha Imlili is relatively very simple in terms of biodiversity and, as such, constitutes a natural laboratory allowing the testing, at least to some extent, of such models. The acanthocephalan population in *C. guineensis* in the Sebkha appears to be stable and fits one of the model patterns suggested by Dobson and Keymer [32]: long term consistency where hosts are present throughout the year and where there is a relatively constant intermediate host density.

In conclusion, fish at the Sebkha Imlili were found to act as definitive hosts for one acanthocephalan and second intermediate hosts for two digeneans. The acanthocephalan population appears to be stable, which indicates the occurrence of regulatory factors given that they live under conditions comparable to hosts held in captivity under otherwise ideal conditions for super infection to occur. Nevertheless, there also appears to be at least two non-exclusive periods of peak maturation for the worms as measured by both total female length and by the proportion of gravid worms found (winter and summer), indicating that there may be some times of the year that are more favorable to transmission, maturation and development than others. While our data do not allow us to state if these relative spikes are the only two periods of high worm maturation because we did not sample continuously throughout the year, they do indicate that there are clearly times of the year when there are relatively more female worms that are smaller and immature (herein spring and fall) and other times in which more females reach maturity (herein winter and summer). This suggests at least some level of cycling in the recruitment and/or development of the worms. Drivers of such a maturation cycle, whether intrinsic or extrinsic, are as yet unknown and further year-round sampling would be required to determine these factors.

The acanthocephalan we found was identified as *A.* (*A.*) cf. *tilapiae*, which appears to have infected *C. guineensis* via host switching from a freshwater tilapia during the Holocene and transitioned to salt water. However, we also entertain the idea that this parasite could be *A. papilio*, which, assuming the latter is a valid species, would have been carried over, with all its hosts (intermediate and definitive) during the Holocene and whose life cycle persisted thanks to the euryhaline character of all involved hosts and their capacity to withstand extreme salinities. Although this is the most parsimonious explanation of the persistence of this acanthocephalan at the Sebkha, the current status of information regarding this species of acanthocephalan is, in our opinion, not reliable and a thorough genetic study of the African *Acanthogyrus* (*Acanthosentis*) species, in particular *A.* (*A.*) *tilapiae*, is called for to determine its origin and to determine if this is a species complex encompassing a much broader distribution than reported thus far (i.e., encompassing marine hosts). Given the simplified ecosystem of the Sebkha, the intermediate host of this acanthocephalan must be one of the two microcrustaceans (harpacticoid copepod or ostracod) that inhabit the water holes and are known prey items of the definitive host. The harpacticoid is the most plausible candidate given that other cycles known for *Acanthosentis* species also incorporate a copepod, including a marine harpacticoid. While such a two-host cycle is rigid, and consequently vulnerable, several factors appear to have allowed this parasite to escape these constraints, including the likelihood of post-cyclic transmission and the fact that both putative intermediate hosts are euryhaline and able to live at very high salinities. Thus, this acanthocephalan illustrates a case of ecological fitting and resilience in an extreme environment [14, 49]. Regarding the digenean *P. genata*, we infer that the first intermediate host must be the hydrobiid snail *E. ventrosa*, because it is the sole gastropod found in the water holes, and that the definitive hosts are likely not omnipresent at the Sebkha given the seasonal pattern of infection. Thus, this digenean, in contrast to the acanthocephalan, could be a recent parasite acquisition for the fish in the Sebkha. It is, nevertheless, as for *A.* (*A.*) *tilapiae,* reportedly a freshwater parasite that owes its survival in the water holes to its low specificity for intermediate hosts with the capacity of living in hypersaline waters. While the persistence of all three parasites in the Sebkha shows the resilience of life in extreme conditions and can project the fate of some parasites in times of climate change, they are nevertheless highly vulnerable as their life cycles would be interrupted if their invertebrate hosts were to disappear.

## References

[1] Agnèse J-F, Louizi H, Berrada Rkhami O, Benhoussa A, Qninba A, Pariselle A. 2020. Des poissons dans le désert : Les Tilapias de la Sebkha d’Imlili, in Sebkhat Imlili (région Dakhla-Oued Eddahab) une zone humide relique. Qninba A, Semlali ML, Pariselle A, Himmi O, Editors. AZ éditions: Rabat. p. 101–106.

[2] Agnèse J-F, Louizi H, Gilles A, Berrada Rkhami O, Benhoussa A, Qninba A, Pariselle A. 2018. A euryhaline fish lost in the desert: The unexpected metapopulation structure of *Coptodon guineensis* (Günther, 1862) in the Sebkha of Imlili. Comptes Rendus Biologies, 341, 75–84. 29402590 10.1016/j.crvi.2018.01.002

[3] Agosta S, Brooks D. 2020. The major metaphors of evolution: Darwinism then and now. Cham, Switzerland: Springer.

[4] Ainou H, Louizi H, Rahmouni I, Pariselle A, Benhoussa A, Rkhami OB, Agnèse J-F. 2021. The discovery of *Coptodon guineensis* (Günther, 1862) (Perciformes, Cichlidae) in the Moulay Bousselham lagoon extends the species’ range 1000 km northward in Morocco. Check List, 7, 1365–1373.

[5] Albecker M, Wilkins L, Krueger-Hadfield S, Bashevkin S, Hahn M, Hare M, Kindsvater H, Sewell M, Lotterhos K, Reitzel A. 2021. Does a complex life cycle affect adaptation to environmental change? Genome-informed insights for characterizing selection across complex life cycle. Proceedings of the Royal Society B: Biological Sciences, 288, 0212122. 10.1098/rspb.2021.2122 PMC8634620 34847763

[6] Altschul SF, Gish W, Miller W, Myers EW, Lipman DJ. 1990. Basic local alignment search tool. Journal of Molecular Biology, 215, 403–410. 2231712 10.1016/S0022-2836(05)80360-2

[7] Amin OM. 1978. Intestinal helminths of some fishes near Cairo, Egypt with redescriptions of *Camallanus kirandensis* Baylis 1928 (Nematoda) and *Bothriocephalus aegyptiacus* Rysavy and Moravec, 1975 (Cestoda). Journal of Parasitology, 64, 93–101. 627980

[8] Amin OM. 1981. The seasonal distribution of *Echinorhynchus salmonis* (Acanthocephala: Echinorhynchidae) among rainbow smelt, *Osmerus mordax* Mitchell, in Lake Michigan. Journal of Fish Biology, 19, 467–474.

[9] Amin OM, Heckmann RA. 2012. An SEM study of *Acanthogyrus* (*Acanthosentis*) *tilapiae* (Acanthocephala: Quadrigypidae) from Africa documenting previously unreported features and host parasite interface. Scientia Parasitologica, 13, 57–63.

[10] Amin OM, Hendrix SS. 1999. Acanthocephala of cichlids (Pisces) in Lake Malawi, Africa, with a description of *Acanthogyrus* (*Acanthosentis*) *malawiensis* sp. n. (Quadrigyridae) from *Labeo cylindricus* Peters, 1852 (Cyprinidae). Journal of Helminthology, 66, 47–55.

[11] Amin OM, Chaudhary A, Heckmann RA, Ha NV, Singh HS. 2019. The morphological and molecular description of *Acanthogyrus* (*Acanthosentis*) *fusiformis* n. sp. (Acanthocephala: Quadrigyridae) from the catfish *Arius* sp. (Ariidae) in the Pacific Ocean off Vietnam, with Notes on Zoogeography. Acta Tropica, 64, 779–796. 10.2478/s11686-019-00102-3 31332657

[12] Amin OM, Heckmann RA, Zargar UR. 2017. Description of a new quadrigyrid acanthocephalan from Kashmir, with notes on metal analysis and histopathology, and a key to species of the subgenus *Acanthosentis* from the Indian subcontinent. Journal of Parasitology, 103, 458–470. 28589837 10.1645/17-27

[13] Anderson GR, Barker SC. 1998. Inference of phylogeny and taxonomy within the Didymozoidae (Digenea) from the second internal transcribed spacer (ITS) of ribosomal DNA. Systematic Parasitology, 41, 87–94.

[14] Araujo S, Braga M, Brooks D, Agosta S, Hoberg E, Von Hartenthal F, Boeger W. 2015. Understanding host-switching by ecological fitting. PLoS ONE, 10, e0139225. 26431199 10.1371/journal.pone.0139225 PMC4592216

[15] Atalabi TE, Awharitoma AO, Akinluyi FO. 2018. Prevalence, intensity, and exposed variables of infection with Acanthocephala parasites of the gastrointestinal tract of *Coptodon zillii* (Gervais, 1848) [Perciformes: Cichlidae] in Zobe Dam, Dutsin-Ma Local Government Area, Katsina State, Nigeria. Journal of Basic and Applied Zoology, 79, 29.

[16] Auld S, Tinsley M. 2015. The evolutionary ecology of complex lifecycle parasites: Linking phenomena with mechanisms. Heredity, 114, 125–132. 25227255 10.1038/hdy.2014.84 PMC4815630

[17] Barson M, Přikrylová I, Vanhove MPM, Huyse T. 2010. Parasite hybridization in African *Macrogyrodactylus* spp. (Monogenea, Platyhelminthes) signals historical host distribution. Parasitology, 137, 1585–1595. 20444301 10.1017/S0031182010000302

[18] Bayed A, Beaubrun PC, Thevenot M, Korgan NC. 1987. Lagunes et marais côtiers du Maroc et de l'Algérie, in African wetlands and shallow water bodies. Burgis NJ, Symoens JJ, Editors. Éditions de l’ORSTOM: Paris, France. p. 35–51.

[19] Baylis HA. 1947. A new acanthocephalan from an east African freshwater fish. Annals and Magazine of Natural History Series, 11, 861–868.

[20] Blakeslee AMH, Haram LE, Altman I, Kennedy K, Ruiz GM, Miller AW. 2020. Founder effects and species introductions: a host versus parasite perspective. Evolutionary Applications, 13, 559–574. 32431736 10.1111/eva.12868 PMC7045715

[21] Bourguet JP. 1986. Contribution à l'étude de *Cletocamptus retrogressus* Schmankevitch, 1875 (Copepoda, Harpacticoida) I. Développement larvaire-stades copépodites. Crustaceana, 51, 66–76.

[22] Bozkov D. 1976. On the postcycle parasitism in helminths and its biological significance. Angewandte Parasitologie, 17, 85–88. 791023

[23] Bozlov D. 1980. Experimental studies on the passage of mature helminths from *Rana ridibunda* Pall to *Bufo viridis* Laur. Khelminthologia, 10, 24–28.

[24] Brooks D, Boeger W. 2019. Climate change and emerging infectious diseases: Evolutionary complexity in action. Current Opinion in Systems Biology, 13, 75–81.

[25] Brooks DR, Hoberg EP. 2008. Darwin’s necessary misfit and the sloshing bucket: the evolutionary biology of emerging infectious diseases. Evolution: Education and Outreach, 1, 2–9.

[26] Bush AO, Lafferty KD, Lotz JM, Shostak AW. 1997. Parasitology meets ecology on its own terms. Journal of Parasitology, 83, 575–583. 9267395

[27] Castresana J. 2000. Selection of conserved blocks from multiple alignments for their use in phylogenetic analysis. Molecular Biology and Evolution, 17, 540–552. 10742046 10.1093/oxfordjournals.molbev.a026334

[28] Chenuil A, Solignac M, Bernard M. 1997. Evolution of the large-subunit ribosomal RNA binding site for protein L23/25. Molecular Biology and Evolution, 14, 578–588. 9159935 10.1093/oxfordjournals.molbev.a025795

[29] Chowhan JS, Gupta NK, Khera S. 1987. On two new species of the genus *Acanthosentis* Verma and Datta, 1929 (Acanthocephala: Quadrigyridae) from freshwater fishes. Research Bulletin (Science) of the Panjab University, 38, 59–65.

[30] Cribb TH, Anderson GR, Adlard RD, Bray RA. 1998. A DNA-based demonstration of a three-host life-cycle for the Bivesiculidae (Platyhelminthes: Digenea). International Journal for Parasitology, 28, 1791–1795. 9846617 10.1016/s0020-7519(98)00127-1

[31] De Arruda VS, Pinto RM, Muniz-Pereira LC. 2001. New host and geographical records for helminths parasites of Ardeidae (Aves, Ciconiiformes) in Brazil. Revista Brasileira de Zoologia, Supplement 1, 225–232.

[32] Dobson AP, Keymer AE. 1986. Life history models, in Biology of the Acanthocephala. Crompton DWT, Nickol BB, Editors. Cambridge University Press. p. 347–384.

[33] Dollfus RP. 1951. Miscellanea helminthologica Maroccana. IIII. Quelques trématodes, cestodes et acanthocéphales. Archives de l’Institut Pasteur du Maroc, 4, 104–229.

[34] Dollfus RP, Golvan Y-J. 1956. Mission M. Blanc-F. d’Aubenton (1954) V-Acanthocéphales de poissons du Niger. Bulletin de l’Institut Français d’Afrique Noire, 18, 1086–1106.

[35] Dzikowski R, Diamant A, Paperna I. 2003. Trematode metacercariae of fishes as sentinels for a changing limnological environment. Diseases of Aquatic Organisms, 55, 145–150. 12911062 10.3354/dao055145

[36] Dzikowski R, Levy MG, Poore MF, Flowers JR, Paperna I. 2004. Use of rDNA polymorphism for identification of Heterophyidae infecting freshwater fishes. Diseases of Aquatic Organisms, 59, 35–41. 15212290 10.3354/dao059035

[37] Emran A, Qninba A, El Balla T, Pariselle A, Rudant JP, Hara F, Hilali M. 2020. Le fonctionnement de la Sebkha d’Imlili dévoilé par les images Radar Palsar. Un reliquat de passé dans le Sahara marocain témoin de la dernière variation climatique dans des conditions géologiques improbables, in Sebkhat Imlili (région Dakhla-Oued Eddahab) une zone humide relique. Qninba A, Semlali ML, Pariselle A, Himmi O, Editors. AZ Éditions: Rabat. p. 19–32.

[38] Frainer A, McKie BG, Amundsen P, Knudsen R, Lafferty KD. 2018. Parasitism and the biodiversity-functioning relationship. Trends in Ecology and Evolution, 33(4), 260–268. 29456188 10.1016/j.tree.2018.01.011

[39] García-Varela M, Nadler SA. 2006. Phylogenetic relationships of Syndermata based on small subunit (SSU) and large subunit (LSU) of rRNA and cytochrome oxidase subunit I gene sequences. Molecular Phylogenetic and Evolution, 40, 61–72. 10.1016/j.ympev.2006.02.010 16574435

[40] Garey RJ, Near TJ, Nonnemacher MR, Nadler SA. 1996. Molecular evidence for Acanthocephala as a subtaxon of Rotifera. Journal of Molecular Evolution, 43, 287–292. 8703095 10.1007/BF02338837

[41] Gautam NK, Misra PK, Saxena AM, Monks S. 2020. Description of *Pallisentis thapari* n. sp. and a re-description of *Acanthosentis seenghalae* (Acanthocephala, Quadrigyridae, Pallisentinae) using morphological and molecular data, with analysis on the validity of the sub-genera of *Pallisentis*. Zootaxa, 4766, 139–156. 10.11646/zootaxa.4766.1.7 33056609

[42] Ghamizi M, Boulaassafer K, Himmi O, Qninba A. 2020. Les Mollusques de la Sebkha d’Imlili, in Sebkhat Imlili (région Dakhla-Oued Eddahab) une zone humide relique. Qninba A, Semlali ML, Pariselle A, Himmi O, Editors. AZ éditions: Rabat. p. 85–92.

[43] Golvan Y-J. 1961. Le phylum des Acanthocephalala. 3éme note : La classe des Palaeacanthocephala (Meyer 1931) (*fin*). Annales de Parasitologie Humaine et Comparée, 36, 717–737. 13706763

[44] Golvan Y-J. 1957. Acanthocéphales des poissons. Exploration hydrobiologique des lacs Kivu, Edouard et Albert (1952–1954). Résultats scientifiques. Institut Royal des Sciences Naturelles de Belgique, 3, 55–64.

[45] Hernández-Orts JS, Brandão M, Georgieva S, Raga JA, Crespo EA, Luque JL, Aznar FJ. 2017. From mammals back to birds: Host-switch of the acanthocephalan *Corynosoma australe* from pinnipeds to the Magellanic penguin *Spheniscus magellanicus*. PLoS ONE, 12(10), e0183809. 28981550 10.1371/journal.pone.0183809 PMC5628790

[46] Hilali M, Baki Benaissi L, Emran A, Bahaj T, Eddahby L. 2020. Caractérisation hydrologique et hydrogéologique d’une sebkha soumise à un climat de type saharien: Cas de la sebkha d’Imlili (Province Oued Eddahab, Maroc), in Sebkhat Imlili (région Dakhla-Oued Eddahab) une zone humide relique. Qninba A, Semlali ML, Pariselle A, Himmi O, Editors. AZ éditions: Rabat. p. 3–18.

[47] Hill-Spanik KM, Sams C, Connors VA, Bricker T, de Buron I. 2021. Molecular data reshape our understanding of the life cycles of three digeneans (Monorchiidae and Gymnophallidae) infecting the bivalve, *Donax variabilis*: It’s just a facultative host!. Parasite, 28, 34. 33835020 10.1051/parasite/2021027 PMC8034251

[48] Himmi O, Bayed A, El Agbani MA, Hara F, El Balla T, Khayya ML, Qninba A. 2020. Biodiversité aquatique de la Sebkha d'Imlili, in Sebkhat Imlili (région Dakhla-Oued Eddahab) une zone humide relique. Qninba A, Semlali ML, Pariselle A, Himmi O, Editors. AZ Éditions: Rabat. p. 71–84.

[49] Hoberg EP, Brooks DR. 2015. Evolution in action: climate change, biodiversity dynamics and emerging infectious disease. Philosophical Transactions of the Royal Society B., 370, 20130553. 10.1098/rstb.2013.0553 PMC4342959 25688014

[50] Huys R, Bodin P. 1997. First record of acanthocephalan in marine copepods. Ophelia, 3, 217–231.

[51] Ibn Tattou M. 2020. Contribution à l'étude de la flore et de la végétation de Sebkhat Imlili (Sahara océanique), in Sebkhat Imlili (région Dakhla-Oued Eddahab) une zone humide relique. Qninba A, Semlali ML, Pariselle A, Himmi O, Editors. AZ Éditions: Rabat. p. 49–62.

[52] Ibrahim MM, Soliman MFM. 2010. Prevalence and site preferences of heterophyid metacercariae in *Tilapia zillii* from Ismalia freshwater canal, Egypt. Parasite, 17, 233–239. 21073146 10.1051/parasite/2010173233

[53] Jensen K, Bullard SA. 2010. Characterization of a diversity of tetraphyllidean and rhinebothriidean cestode larval types, with comments on host associations and life-cycles. International Journal for Parasitology, 40, 889–910. 20026125 10.1016/j.ijpara.2009.11.015

[54] Katoh K, Standley DM. 2013. MAFFT Multiple Sequence Alignment Software Version 7: Improvements in Performance and Usability. Molecular Biology and Evolution, 30, 772–780. 23329690 10.1093/molbev/mst010 PMC3603318

[55] Kennedy CR. 2006. Ecology of the Acanthocephala. Cambridge Univ. Press: New York. p. 249.

[56] Khalil LF. 1971. Check-list of the helminth parasites of African freshwater fishes. Commonwealth Agricultural Bureau: Slough, UK.

[57] Kidé NG, Dunz A, Agnèse J-F, Dilyte J, Pariselle A, Carneiro C. 2016. Cichlids of the Banc d’Arguin National Park, Mauritania: insight into the diversity of the genus *Coptodon*. Journal of Fish Biology, 88, 1369–1393. 26856797 10.1111/jfb.12899

[58] Koch RW, Shannon RP, Detwiler JT, Bolek MG. 2021. Molecular identification of juvenile *Neoechinorhynchus* spp. (Phylum: Acanthocephala) infecting ostracod and snail hosts provides insight into acanthocephalan host use. Journal of Parasitology, 107, 739–761. 34546335 10.1645/20-130

[59] Køie M. 1990. *Pygidiospis ardae* n.sp. (Digenea: Heterophyidae: Pygidiopsinae) in the grey Heron *Ardea cinerea* L. from Denmark. Systematic Parasitology, 15, 141–149.

[60] Lassiere OL. 1988. Host-parasite relationships between larval *Sialis lutaria* (Megaloptera) and *Neoechinorhynchus rutili* (Acanthocephala). Parasitology, 97, 331–338. 3200605 10.1017/s0031182000058522

[61] Lemoine F, Correia D, Lefort V, Doppelt-Azeroual O, Mareuil F, Cohen-Boulakia S, Gascuel O. 2019. NGPhylogeny.fr: new generation phylogenetic services for non-specialists. Nucleic Acids Research, 47, W260–W265. 31028399 10.1093/nar/gkz303 PMC6602494

[62] Louizi H, Agnèse JF, Bitja Nyom A, de Buron I, Berrada Rkhami O, Benhoussa A, Qninba A, Pariselle A. 2019. The distribution and systematic status of cichlid fishes (Teleostei, Cichliformes: Cichlidae) from Morocco. Vie et milieu – Life and Environment, 69, 95–106.

[63] Louizi H, Vanhove MPM, Rahmouni I, Berrada Rkhami O, Benhoussa A, Van Steenberge M, Pariselle A. 2022. Species depauperate communities and low abundances of monogenean gill parasites at the edge of the natural distribution range of their cichlid hosts in northern Africa. Hydrobiologia. Advances in Cichlid Research V, 1, 1–11.

[64] Luton K, Walker D, Blair D. 1992. Comparisons of ribosomal internal transcribed spacers from two congeneric species of flukes (Platyhelminthes: Trematoda: Digenea). Molecular and Biogeochemical Parasitology, 56, 323–328. 10.1016/0166-6851(92)90181-i 1484553

[65] Mahdy OA, Abdel-Maogood SZ, Abdelsalam M, Shaalan M, Abdelrahman HA, Salem MA. 2021. Epidemiological study of fish-borne zoonotic trematodes infecting Nile tilapia with first molecular characterization of two heterophyid flukes. Aquaculture Research, 52, 4475–4488.

[66] Marchand B. 1984. A comparative ultrastructural study of the shell surrounding the mature acanthor larvae of 13 acanthocephalan species. Journal of Parasitology, 70, 886–901.

[67] Mason G. 2010. Species differences in responses to captivity: Stress, welfare and the comparative method. Trends in Ecology & Evolution, 25, 713–721. 20952089 10.1016/j.tree.2010.08.011

[68] Meddour A, Meddour BK, Brahim-Tazi NA, Zouakh DE, Mehennaoui S. 2010. Microscopie électronique à balayage des parasites des poissons du lac Oubeira Algérie. European Journal of Scientific Research, 48, 129–141.

[69] Mediani M, El Mouden E, Slimani T, Qninba A. 2020. Reptiles de la Sebkha d’Imlili (Sahara Atlantique Marocain): État des lieux et perspectives de conservation, in Sebkhat Imlili (région Dakhla-Oued Eddahab) une zone humide relique. Qninba A, Semlali ML, Pariselle A, Himmi O, Editors. AZ Éditions: Rabat. p. 113–116.

[70] Menasria A, Barčák D, Kaouachi N, Bensouilah M, Scholz T, Hernández-Orts JS. 2019. Redescription of *Acanthogyrus* (*Acanthosentis*) *maroccanus* (Dollfus, 1951) (Acanthocephala: Quadrigyridae), a parasite of the Algerian barb *Luciobarbus callensis* (Valenciennes) (Cyprinidae) in Algeria, and first molecular data. Journal of Helminthology, 94(e82), 1–12. 10.1017/S0022149X19000737 31466552

[71] Mhammdi N, Cheddadi R, Slimani H, Qninba A. 2020. Histoire Holocène de la sebkha d’Imlili (Sahara Marocain), in Sebkhat Imlili (région Dakhla-Oued Eddahab) une zone humide relique. Qninba A, Semlali ML, Pariselle A, Himmi O, Editors. AZ Éditions: Rabat. p. 41–48.

[72] Mielke W. 2000. *Cletocamptus retrogressus* (Copepoda, Harpacticoida) from irrigation and drainage ditches of the Rhone delta (Camargue, France): a redescription. Vie et Milieu, 51, 1–9.

[73] Mohd-Agos S, Mohd-Husin N, Zakariah MI, Yusoff NAH, Wahab W, Jones JB, Hassan M. 2021. Three new species of Acanthocephala from *Acanthogyrus* (*Acanthosentis*) (Acanthocephala: Quadrigyridae) from tinfoil barb fish, *Barbonymus schwanenfeldii* in Lake Kenyir, Terengganu, Malaysia. Tropical Biomedicine, 38, 387–395. 34608112 10.47665/tb.38.3.064

[74] Muzzal PM, Bullock WL. 1978. Seasonal occurrence and host-parasite relationships of *Neoechinorhynchus saginatus* VanCleave and Bangham, 1949 in the fallfish *Semotilus corporalis* (Mitchell). Journal of Parasitology, 64, 860–865.

[75] Nachite D, Rodríguez-Lázaro J, Rubio MM, Pascual A, Bekkali R. 2010. Distribution and ecology of recent ostracods from the Tahadart estuary (NW Morocco) / Distribution et écologie des associations d’ostracodes récents de l’estuaire de Tahadart (Maroc Nord-Occidental). Revue de Micropaléontologie, 53, 3–15.

[76] Nickol BB. 1985. Epizootiology, in Biology of the Acanthocephala. Crompton DWT, Nickol BB, Editors. Cambridge University Press: Cambridge, United Kingdom. p. 307–346.

[77] Nickol BB. 2003. Is postcyclic transmission underestimated as an epizotiological factor for acanthocephalans? Helminthologia, 40, 93–95.

[78] Okamura B, Gruhl A, de Baets K. 2022. Evolutionary transitions of parasites between freshwater and marine environments. Integrative and Comparative Biology, 62(2), 345–356. 35604852 10.1093/icb/icac050

[79] Paperna I, Dzikowski R. 2006. Digenea (Phylum Platyhelminthes), in Fish Diseases and Disorders. Vol. Protozoan and Metazoan Infections. Woo PTK, Editor. CAB International: Wallingford, United Kingdom. p. 345–390.

[80] Qninba A, Mataame A. 2009. Mise au point sur la répartition au Maroc des Cichlidés (Pisces, Perciformes) basée sur les échantillons conservés dans les collections du Muséum national d’Histoire naturelle de l’Institut Scientifique (Rabat, Maroc). Bulletin de l’Institut Scientifique, Rabat, Section Sciences de La Vie, 31, 57–61.

[81] Qninba A, Benhoussa A, Samlali ML, Pariselle A, Agnèse J-F, de Buron I. 2017. Observations ornithologiques du 14 au 16 avril 2017 à la Sebkha d’Imlili (Sud marocain). Go-South Bulletin, 14, 37–42.

[82] Qninba A, El Agbani MA, Radi M, Pariselle A. 2012. Sur la présence de *Tilapia guineensis* (Teleostei, Cichlidae) dans les gueltas d’un affluent de l’Oued Chbeyka, l’oued Aabar (Province de Tan Tan, Sud-Ouest du Maroc). Bulletin de l’Institut Scientifique, Rabat, Section Sciences de La Vie, 34, 125–126.

[83] Qninba A, El Brini H, Radi M, Mhimdate H, Samlali ML, Khayya ML. 2020. Inventaire commenté des Mammifères des environs de la Sebkha d’Imlili, in Sebkhat Imlili (région Dakhla-Oued Eddahab) une zone humide relique. Qninba A, Semlali ML, Pariselle A, Himmi O, Editors. AZ Éditions: Rabat. p. 127–134.

[84] Qninba A, Ibn Tattou M, Radi M, El Idrissi A, Essougrati H, Bensouiba H, Ben Moussa S, Ougga T, Bouzrou J, Azaguagh I, Bensbai J, Khayya ML. 2009. Sebkhet Imlily, une zone humide originale dans le Sud marocain. Bulletin de l’Institut Scientifique, Rabat, section Sciences de la Vie, 31, 51–55.

[85] Qninba A, Semlali ML, Pariselle A, Himmi O. 2020. Sebkhat Imlili (région Dakhla-Oued Eddahab) une zone humide relique. AZ Éditions: Rabat. p. 140.

[86] Radi M, El Idrissi Essougrati A, El Balla T, El Agbani MA, Samlali ML, Qninba A. 2020. Inventaire commenté des oiseaux de Sebkhat Imlili et de ses environs immédiats, in Sebkhat Imlili (région Dakhla-Oued Eddahab) une zone humide relique. Qninba A, Semlali ML, Pariselle A, Himmi O, Editors. AZ Éditions: Rabat. p. 117–126.

[87] Ramdani M. 1988. Les eaux stagnantes du Maroc, études biotypologiques et biogéographiques du Zooplancton. Travaux de l’Institut Scientifique de Rabat, Zoologie, 43, 40 p.

[88] Rjimati E, Zemmouri A. 2011. Carte Géologique du Maroc au 1/50 000 Feuille Oum Tlayha, Notice Explicative, Notes et Mémoires du Service Géologique N° 510 bis : Rabat. p. 94.

[89] Ru S-S, Rehman AU, Chen H-X, Suleman Khan MS, Muhammad N, Li L. 2022. Morphology and molecular characterization of *Acanthogyrus* (*Acanthosentis*) *bilaspurensis* Chowhan, Gupta & Khera, 1987 (Acanthocephala: Gyracanthocephala: Quadrigyridae) from the common carp *Cyprinus carpio* Linnaeus (Cypriniformes: Cyprinidae) in Pakistan. Parasitology International, 90, 102608. 35680008 10.1016/j.parint.2022.102608

[90] Schmidt GD. 1985. Development and life cycles, in Biology of the Acanthocephala. Crompton DWT, Nickol BB, Editors. Cambridge University Press: Cambridge, United Kingdom. p. 273–305.

[91] Sharifdini M, Amin OM, Heckman RA. 2021. The molecular profile of *Acanthogyrus (Acanthosentis) kashmirensis* from the Indian subcontinent. Acta Parasitologica, 66, 863–870. 33609238 10.1007/s11686-020-00331-x

[92] Sharma SK, Wattal BL. 1976. First record of a cyclopoid host- *Mesocyclops leuckarti* (Clauss) for an acanthocephalan worm – *Acanthosentis dattai* Podder from Delhi (India). Folia Parasitologica, 23, 169–173. 1278821

[93] Song R, Li WX, Wu SG, Zou H, Wang GT. 2014. Population genetic structure of the acanthocephalan *Acanthosentis cheni* in anadromous, freshwater, and landlocked stocks of its fish host, *Coilia nasus*. Journal of Parasitology, 100, 193–197. 24224788 10.1645/12-144.1

[94] Stiassny MJ, Lamboj A, De Weirdt D, Teugels GG. 2008. Cichlidae, in The fresh and brackish water fishes of Lower Guinea, West-Central Africa. Institut de Recherche pour le Développement. Volume 2. Collection faune et flore tropicales 42. Stiassny MLJ, Teugels GG, Hopkins CD, Editors. Institut de Recherche pour le Développement: Paris, France. Muséum national d’histoire naturelle, Paris, France et Musée royal de l’Afrique Centrale, Tervuren, Belgium. p. 269–403.

[95] Tamura K, Stecher G, Kumar S. 2021. MEGA11: Molecular Evolutionary Genetics Analysis version 11. Molecular Biology and Evolution, 38, 3022–3027. 33892491 10.1093/molbev/msab120 PMC8233496

[96] Tkach VV, Littlewood DTJ, Olson PD, Kinsella JM, Swiderski Z. 2003. Molecular phylogenetic analysis of the Microphalloidea Ward, 1901 (Trematoda: Digenea). Systematic Parasitology, 56, 1–15. 12975618 10.1023/a:1025546001611

[97] Troncy PM. 1970. Contribution à l’étude des helminthes d’Afrique, principalement du Tchad. Bulletin du Muséum National d’Histoire Naturelle, 41, 1487–1511.

[98] Troncy PM. 1974. Acanthocéphales parasites de poissons du Tchad. Note de synthèse, in Proceedings of the 3rd International Congress of Parasitology, Munich III (Section G2), 1622 p.

[99] Troncy PM, Vassiliadès G. 1974. Acanthocéphales parasites de poissons d’Afrique. Bulletin de l’Institut Fondamental d’Afrique Noire, 36, 902–907.

[100] Uznanski RL, Nickol BB. 1980. Parasite population regulation: lethal and sublethal effects of *Leptorhynchoides thecatus* (Acanthocephala: Rhadinorhynchidae) on *Hyalella azteca* (Amphipoda). Journal of Parasitology, 66, 121–126.

[101] Yahyaoui A, Perea S, Garzon P, Doadrio I. 2020. Atlas des poissons des eaux continentales du Maroc. FSR-MNCNM.

[102] Ye J, Coulouris G, Zaretskaya I, Cutcutache I, Rozen S, Madden T. 2012. Primer-BLAST: A tool to design target-specific primers for polymerase chain reaction. BMC Bioinformatics, 13, 134. 22708584 10.1186/1471-2105-13-134 PMC3412702

